# Synergistic tumor microenvironment modulation enabled by a nanozyme-boosted biomimetic macrophage-derived nanovesicle for highly efficient antitumor therapy

**DOI:** 10.7150/thno.114467

**Published:** 2025-07-11

**Authors:** Yue Su, Haibin Wu, Ke Duan, Jiahao Xie, Weitao Huang, Xiaozhou Mou, Xiangming Ye, Yeyu Shen, Ting Li, Junjia He, Luoqin Fu, Yin Wang, Liping Wen, Qiong Bian, Mingang Zhu, Xiangmin Tong

**Affiliations:** 1 Center for Rehabilitation Medicine, Rehabilitation and Sports Medicine Research Institute of Zhejiang Province, Department of Rehabilitation Medicine, Zhejiang Provincial People's Hospital, Affiliated People's Hospital, Hangzhou Medical College, Hangzhou, Zhejiang 310014, China.; 2 Clinical Research Institute, Zhejiang Provincial People's Hospital, Affiliated People's Hospital, Hangzhou Medical College, Hangzhou, Zhejiang 310014, China.; 3 School of Pharmaceutical Sciences, Hangzhou Medical College, Hangzhou 311399, Zhejiang, China.; 4 Center for Plastic & Reconstructive Surgery, Department of Dermatology, Zhejiang Provincial People's Hospital, Affiliated People's Hospital, Hangzhou Medical College, Hangzhou, Zhejiang 310014, China.; 5 Department of Dermatology, the First People's Hospital of Jiashan, Jiashan Hospital Affiliated to Jiaxing University, Jiaxing 314100, Zhejiang, China.; 6 Department of Urology, the First People's Hospital of Fuyang District, Hangzhou, Zhejiang 311400, China.; 7 Department of Hematology, the Affiliated Hangzhou First People's Hospital, Westlake University School of Medicine, Hangzhou, 310006, China.

**Keywords:** tumor microenvironment, nanozymes, biomimetic macrophage-derived nanovesicles, co-extrusion technology, homologous targeting

## Abstract

**Rationale:** The tumor microenvironment (TME) plays a pivotal role in cancer progression, with tumor-associated macrophages (TAMs) serving as key contributors. Immunosuppressive M2-type TAMs are associated with poor prognosis and treatment resistance, highlighting the need for strategies to reprogram these cells into pro-inflammatory M1 phenotypes. To address this, we developed a TME-reshaping nanoplatform combining the tumor-targeting capability of M1 macrophage-derived nanovesicles (M1NVs) with the immunomodulatory and catalytic properties of hollow, virus-spiky hMnO_x_ nanozymes. This approach aims to enhance chemotherapy delivery while simultaneously reversing immunosuppression and boosting antitumor immunity.

**Methods:** We engineered a biomimetic nanoplatform by physically co-extruding M1NVs with hMnO_x_ nanozymes. The platform was evaluated in a malignant melanoma model characterized by M2 TAM infiltration, using the first-line chemotherapeutic agent dacarbazine (DTIC) as a model drug. The system's tumor-targeting ability, cytotoxicity, and immunomodulatory effects were assessed. Additionally, the capacity of hMnO_x_ nanozymes to induce immunogenic cell death (ICD) and promote antigen presentation was investigated.

**Results:** The nanoplatform demonstrated precise tumor-targeted delivery of DTIC *via* M1NVs, effectively inducing tumor cell death. The combination of M1NVs and hMnO_x_ nanozymes successfully repolarized M2 TAMs into pro-inflammatory M1 macrophages, alleviating immunosuppression and enhancing immunotherapy efficacy. Furthermore, hMnO_x_ nanozymes triggered ICD and improved antigen presentation, amplifying antitumor immune responses. The fabrication process was simple and scalable, underscoring the platform's potential for clinical translation.

**Conclusion:** This study presents a novel nanozyme-boosted biomimetic macrophage-derived nanovesicle system that integrates precise tumor targeting, chemotherapy delivery, and TME immunomodulation. By repolarizing TAMs and enhancing antitumor immunity, the platform offers a promising strategy to overcome treatment resistance in immunosuppressive tumors. Its scalable production and high clinical potential make it a viable candidate for future cancer therapy applications.

## Introduction

One of the biggest threats to world health in the twenty-first century is cancer. According to the Global Cancer Report 2022, cancer ranks as the first or second leading cause of mortality for individuals under seventy years old in 177 countries worldwide. One of the most pressing issues facing public health today is cancer treatment 1. Tumor microenvironment (TME) is a highly structured ecosystem that provides an important ecological niche for cancer development and progression. Tumor-targeted therapy and improvement of the tumor microenvironment have been the hotspots of tumor therapy 2-4.

Tumor-associated macrophages (TAMs) represent a critical element within the TME and are frequently linked to unfavorable clinical outcomes, as well as resistance to various therapeutic approaches, including immunotherapy 5. TAMs are classified according to their function as classically activated state macrophages (M1) and alternatively activated macrophages (M2). M1 TAMs have the ability to produce various pro-inflammatory factors, including TNF-α, IL-12, IL-23, IL-1β, and IL-6. These factors contribute to the promotion of a Th1 (cytotoxic) immune response, which helps inhibit tumor growth. On the contrary, M2 TAMs mainly accelerate cancer progression by inducing immunosuppression, promoting tumor growth and metastasis, and promoting angiogenesis in various ways 6. In the complex tumor microenvironment, macrophages are recruited to the tumor site and can undergo polarization into two distinct states: the M1 TAMs, which suppresses tumor progression, or the M2 TAMs, which facilitates tumor growth 7-9. Macrophage polarization is a highly dynamic process that is capable of switching between the M1 and M2 phenotypes under a variety of conditions 10. More and more studies have been conducted on macrophages as a potential target for tumor immunotherapy, attempting to achieve antitumor effects by inducing the transformation of TAMs from immunosuppressive to immune-promoting types 5,11,12. Therefore, promoting the conversion of M2 TAMs to M1 TAMs in the TME is expected to synergize tumor immunotherapy by stimulating intrinsic autoimmunity.

In recent years, researchers have used membrane-encapsulated and extracellular vesicles (EVs) technologies to achieve tumor targeting and to improve the tumor microenvironment, with a significant increase in interest in the use of extracellular vesicles as potential therapeutic agents 13,14. EVs are membranous vesicles secreted by cells into the extracellular space. During the secretion process, nucleic acids and proteins are encapsulated inside the vesicles, which not only inherits the properties and functions of the parental cells, but also avoids the ethical and safety issues associated with cell therapy 15. However, the number of exosomes secreted by cells is extremely limited and their purification is cumbersome, so researchers have attempted to prepare cell-derived nanovesicles (NVs) by physical extrusion. It was confirmed that NVs obtained by physical extrusion have similar morphological characteristics to cell-secreted exosomes, but their molecular characteristics are more similar to those of the cells themselves, with good biocompatibility, a simple and rapid preparation method, and a high yield 16,17. Using such vesicles, Schweer *et al.* encapsulated chemotherapeutic agents in macrophage-derived NVs and exploited their tumor-site homing properties for precise tumor targeting 18. In addition, researchers have found that M1 macrophage-derived NVs (M1NVs) can not only target the tumor site to deliver drugs, but also influence the polarization of TAMs during their internalization, as Choo *et al.* were able to use M1NVs to repolarize M2 TAMs into M1 TAMs to regulate the TME 19-21. In conclusion, M1NVs were obtained by physical extrusion preparation, which is expected to achieve precise tumor targeting and exert its own polarization-regulating ability to act on TAMs in the tumor microenvironment.

Recent studies have demonstrated that elemental manganese plays a significant role in regulating the polarization of macrophages. Manli Song *et al.* 22. found that HA-modified MnO nanoparticles induced the conversion of M2 TAMs into pro-inflammatory M1 macrophages in the TME, which exerted an effective antitumor effect. Moreover, manganese ions play a crucial role in boosting the functionality of host antigen-presenting cells (APCs), including dendritic cells (DCs) and macrophages. They enhance the presentation of tumor antigens, facilitate the recruitment of cytotoxic T cells into tumor sites, and amplify the targeted destruction of tumor cells by these immune effectors 23,24. We have synthesized a hMnO_x_ nanozymes with an internal hollow structure that facilitates the loading of multiple antitumor drugs 25.

**Figure 1:**
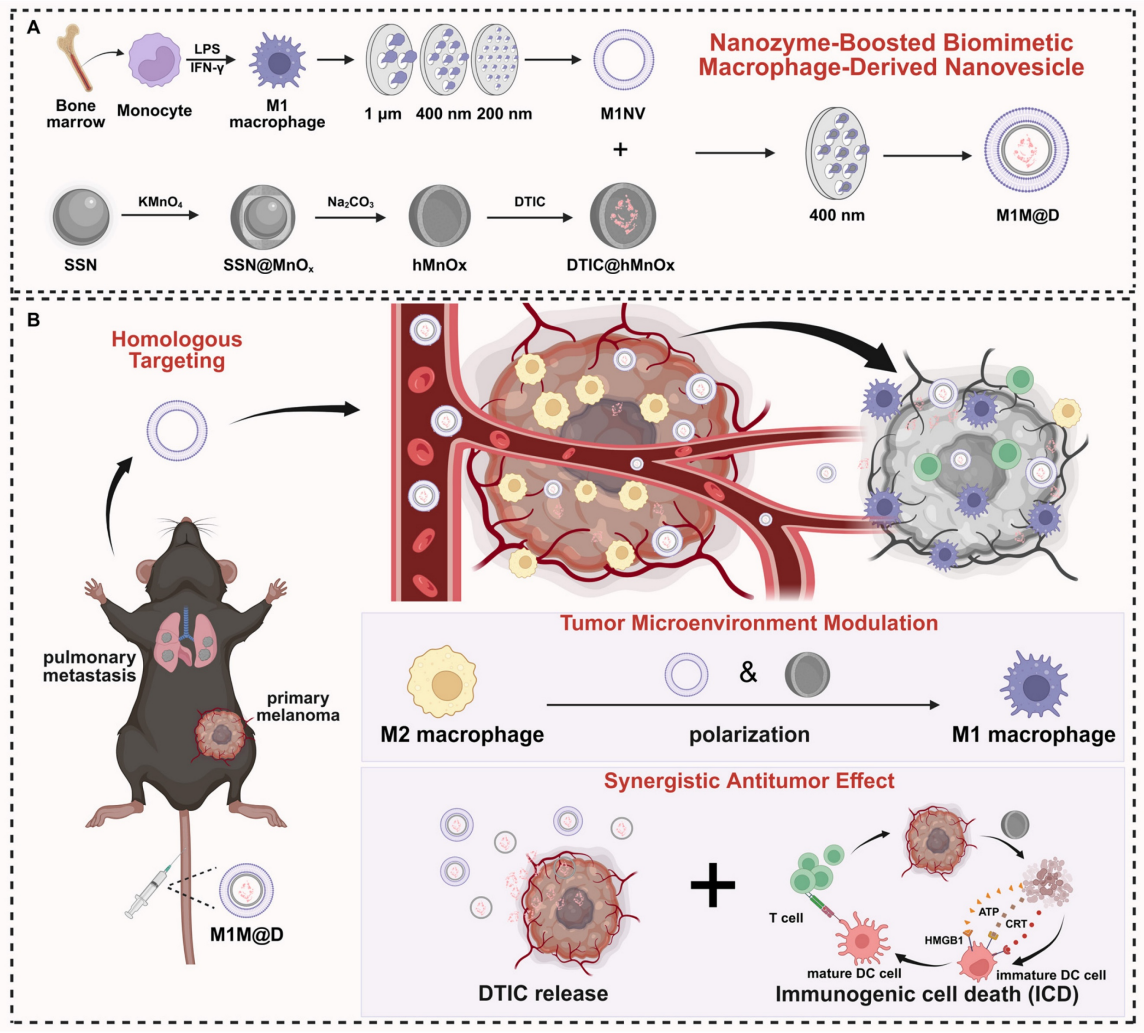
A schematic illustration depicting the fabrication of the M1M@D nanoplatform and its utilization in cancer treatment. (A) The fabrication process of M1M@D. (B) M1M@D was administered to tumor sites through tail vein injection, showing therapeutic effectiveness in both subcutaneous tumor and lung metastasis models. The study also explored its impact on the tumor microenvironment and its ability to enhance the combined antitumor response in tumor tissues.

In this study, we developed an innovative nanozyme-boosted biomimetic macrophage-derived TME reshaping nanoplatform by physically co-extruding M1 macrophage-derived NVs (M1NVs) with hollow, virus-spiky manganese oxide (hMnO_x_) nanozymes. This nanoplatform was specifically evaluated in a malignant melanoma model characterized by significant M2 tumor-associated macrophage (TAM) infiltration, using dacarbazine (DTIC), a first-line chemotherapeutic agent, as the model drug. The resulting M1 macrophage-derived nanovesicle loaded with DTIC and hMnO_x_ nanozyme (M1M@D) features a unique three-layer structure: an outer layer of M1NVs obtained through physical extrusion, a middle layer of hMnO_x_ nanozyme, and an inner core of encapsulated DTIC. Our findings demonstrate that this platform offers a novel immunotherapy strategy with three key mechanisms of action: (1) M1M@D achieves precise tumor targeting through the vesicular homology of M1NVs, enabling localized release of DTIC for effective tumor cell killing; (2) The synergistic action of M1NVs and hMnO_x_ nanozymes reprograms TAMs in the tumor microenvironment by converting immunosuppressive M2 TAMs to pro-inflammatory M1 phenotype, thereby reversing the immunosuppressive state; (3) hMnO_x_ nanozymes facilitate immunogenic cell death (ICD) of tumor cells while promoting dendritic cell (DC) maturation and antigen presentation, ultimately enhancing T-cell-mediated antitumor immunity. The developed nanoplatform demonstrates several advantages for clinical translation: it features a straightforward fabrication process, high production yield, and excellent scalability for mass production. These characteristics, combined with its dual functionality in precision drug delivery and immunomodulation, make this nanoplatform particularly attractive for future translational research and clinical applications in cancer immunotherapy (Figure 1).

This study marks a notable breakthrough in targeted cancer therapy, introducing a versatile strategy that concurrently tackles tumor cell eradication and tumor microenvironment regulation, paving the way for innovative approaches in combination cancer immunotherapy.

## Results and Discussion

### Fabrication and characterization of M1M@Ds

**Figure 2:**
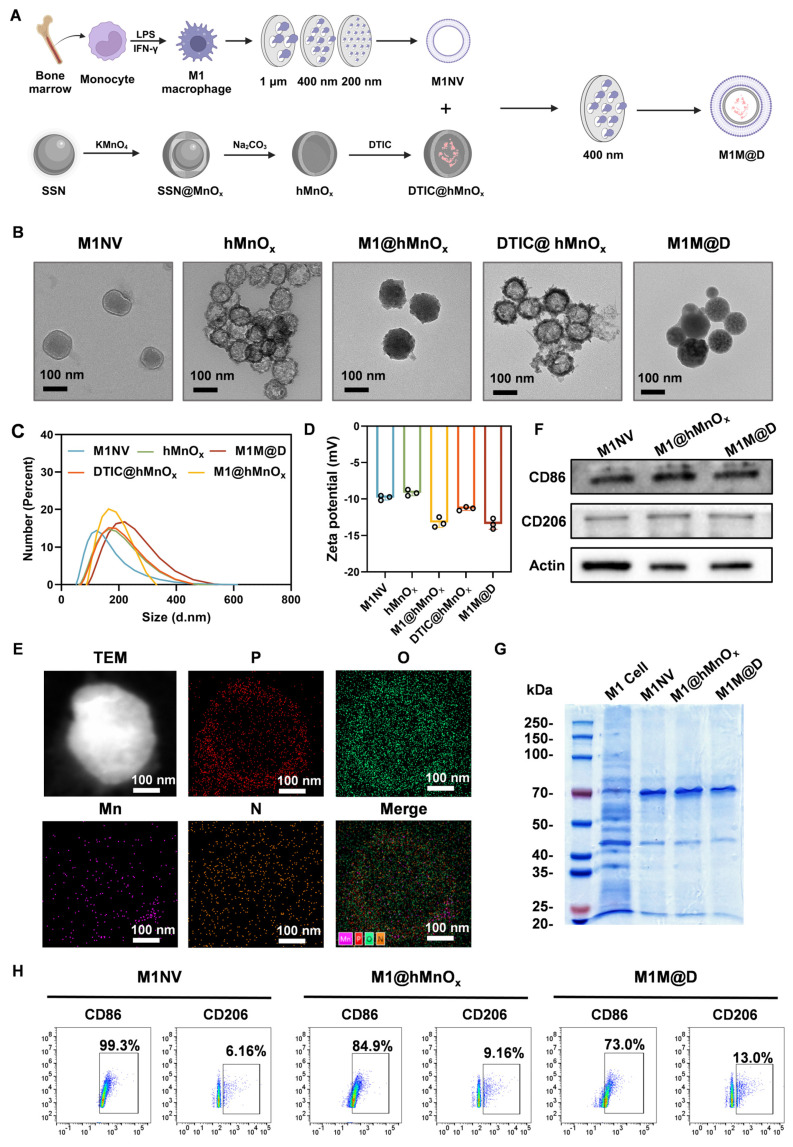
Fabrication and characterization of the M1M@Ds. **(A)** Schematic diagram of M1M@P preparation method **(B)** TEM image of M1NVs, hMnO_x_s, M1@hMnO_x_s, DTIC@hMnO_x_s, and M1M@Ds. **(C)** Particle size distribution of M1NVs, hMnO_x_s, M1@hMnO_x_s, DTIC@hMnO_x_s, and M1M@Ds. **(D)** Zeta potentials of M1NVs, hMnO_x_s, M1@hMnO_x_s, DTIC@hMnO_x_s, and M1M@Ds.** (E)** EDS mapping of M1M@Ds. **(F)** Western blot of M1NVs, M1@hMnO_x_s, and M1M@Ds for detection of CD86 and CD206.** (G)** Coomassie Blue staining of M1 cell, M1NVs, M1@hMnO_x_s, and M1M@Ds.** (H)** Nanoflow of M1NVs, M1@hMnO_x_s, and M1M@Ds for detection of CD86 and CD206.

To prepare M1NVs, we first needed to obtain stable M1 macrophages. By trying a variety of protocols (Figure S1), we finally used the induction of Figure S2. to obtain stable M1 macrophages. Induced M1 macrophages were then identified by flow cytometry using a monoclonal antibody to CD86, a glycoprotein known to be present on the cell membrane of antigen-presenting cells such as blood monocytes and macrophages. The assay showed that the cells expressed CD86 more than 95% (Figure S3). Next, the induced M1 macrophages were collected and centrifuged, resuspended in PBS, and nanoscale vesicular M1NVs were obtained by passing them through polycarbonate membranes with pore sizes of 1 µm, 400 nm, and 200 nm using an extruder apparatus. Nanoflow assay revealed that 1×10^5^ particles/mL M1 macrophages can produce 2.125×10^10^ particles/mL M1NVs by physical extrusion. Then, to confirm the drug-carrying capacity of DTIC@hMnO_x_s, We took the supernatant of DTIC@hMnO_x_s after centrifugation (11000 ×g, 30 min) and then measured the UV absorbance by UV spectrophotometer (UV-2600) (Figure S4). By calculation, we found that the drug loading rate of DTIC@hMnO_x_ was 31.25%. M1M@Ds were then obtained by passing through polycarbonate membranes with pore size of 400 nm using an extruder according to the ratio of M1NVs:DTIC@hMnO_x_s = 100:1 (Figure 2A).

When particles/molecules are physically extruded, the applied pressure forces the identically charged particles to break through the electrostatic repulsion barrier and enter the short-range range of action (typically <2 nm) where van der Waals forces or hydrophobic interactions dominate. Our physical extrusion can force the negatively charged M1NVs and DTIC@hMnO_x_s to temporarily approach each other to form a stable structure. When DTIC@hMnO_x_s is wrapped into M1NVs, the positive charge inside M1NVs and negatively charged DTIC@hMnO_x_s will form a stable concentric circle structure. In order to determine the shape and morphology, observations were made by means of transmission electron microscopy (TEM). Upon inspection, M1NVs was circular in shape, hMnO_x_s, and DTIC@hMnO_x_s showed a bacteriophage-like hollow morphology with porous and rough surfaces, and M1@hMnO_x_s and M1M@Ds were concentric circles, both with smooth surfaces (Figure 2B). To characterize the vesicle size distribution during preparation, we quantified vesicle diameters and potentials. Dynamic light scattering (DLS) analysis revealed that the size of M1NVs were ~122.4 nm, hMnO_x_s were ~159.1 nm, M1@hMnO_x_s were ~164.2 nm, DTIC@hMnO_x_s were ~ 160.1 nm, M1M@Ds were ~ 219.1 nm (Figure 2C); zeta potentials were -9 mV for M1NVs, -9 mV for hMnO_x_s, -13 mV for M1@hMnO_x_s, -11 mV for DTIC@hMnO_x_s, and -13 mV for M1M@Ds (Figure 2D). In addition, we measured the elemental composition of M1M@Ds by energy dispersive spectroscopy (EDS), and the results showed that M1M@Ds contains the elemental compositions of M1NVs and DTIC@hMnO_x_s (Figure 2E).

We tested the stability of M1M@Ds by incubating them in PBS, DMEM and FBS for 7 consecutive days. PBS is utilised for fundamental research to regulate variables, FBS simulates protein interactions in a living organism, and DMEM more closely matches the conditions of cellular experimental procedures. We judged the degree of aggregation by monitoring the change in vesicle size over time. The size of M1M@Ds remained relatively constant for the first 2 days, indicating that M1M@Ds remained stable during this time. After 48 h, the stability gradually decreased, as evidenced by an increase in the size of the vesicles (Figure S5).

To further verify that the nanovesicles synthesized in this study maintained the protein markers on the cell membrane, the expression of cell membrane surface proteins in M1NVs, M1@hMnO_x_s, and M1M@Ds was assessed using Western blot, Coomassie Blue Staining, and Nanoflow analysis. The results of Coomassie Blue Staining showed that M1 cell, M1NVs, M1@hMnO_x_s, and M1M@Ds had similar protein bands (Figure 2G), indicating that the M1NVs were successfully encapsulated and that the integrity of cell membrane proteins was not compromised during the preparation process. The Western blot results showed that M1NVs, M1@hMnO_x_s, and M1M@Ds expressed high levels of CD86 (Figure 2F), which was also validated by Nanoflow (Figure 2H).

We successfully prepared M1M@Ds and characterized them by various methods. The results showed that the prepared nanomimetic multilayered nanovesicles not only conformed to expectations in morphology and size, but also effectively retained the characteristics of cell membrane proteins. These findings provided an important theoretical foundation and experimental basis for the application of M1M@Ds in drug delivery and immunotherapy.

### M1M@D uptake by B16F10 tumor cells and repolarizing M2 macrophages

**Figure 3:**
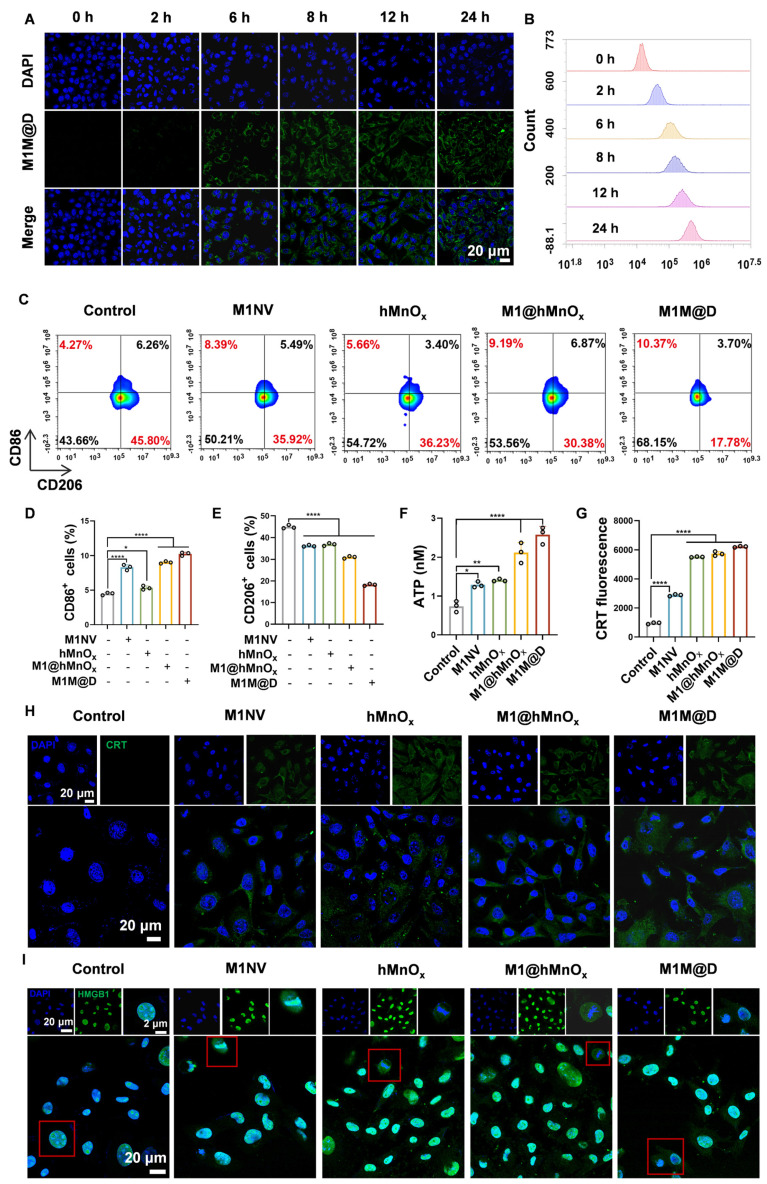
Cellular uptake of M1M@Ds, their effects on repolarize M2 macrophages, and ICD in B16F10 tumor cells.** (A)** The cellular uptake of M1M@Ds was assessed using confocal microscopy and **(B)** FCM analysis. **(C)** FCM of the effects of M1NVs, hMnO_x_s, M1NV@hMnO_x_s, and M1M@Ds on polarization of M2 macrophages** (D)** Changes in cellular CD86 expression. **(E)** Changes in cellular CD206 expression. **(F)** ATP secretion of B16F10 tumor cells treated with M1M@Ds. **(H)** CRT exposure was evaluated through confocal microscopy observations and **(G)** flow cytometry analysis. **(I)** Confocal microscopy images demonstrated the relocation of HMGB1 from the nucleus to the cytoplasm in B16F10 tumor cells after M1M@Ds treatment. All data are expressed as mean ± SD (n = 3). The nuclei of the cells were labeled using DAPI, which is blue. * p < 0.05, ** p < 0.01, *** p < 0.001, **** p < 0.0001.

To test whether M1M@Ds could be taken up by B16F10 tumor cells, we labeled M1M@Ds with the fluorescent dye Dio, and M1M@Ds was added to B16F10 tumor cells for co-culture at 0, 2, 6, 8, 12, and 24 h, respectively. The cellular uptake of M1M@Ds by B16F10 tumor cells was examined using flow cytometry (FCM) and confocal laser scanning microscopy (CLSM). Both CLSM and FCM analyses demonstrated that B16F10 tumor cells effectively internalized M1M@Ds (Figure 3A, B, S6). Similarly, we evaluated the cellular uptake of M1NVs and M1@hMnO_x_s by B16F10 tumor cells. Both CLSM and FCM analyses demonstrated that B16F10 tumor cells effectively internalized M1NVs and M1@hMnO_x_s (Figure S7, S8).

Next, we tested whether M1M@Ds could promote M2 macrophage repolarization to proinflammatory phenotype. Changes in the expression of CD86^+^ and CD206^+^ in M2 macrophages alone and in M2 macrophages co-incubated with the addition of M1NVs, hMnO_x_s, M1@hMnO_x_s, and M1M@Ds were compared. The greatest increase in CD86^+^ and decrease in CD206^+^ was found by flow cytometry using M1M@Ds (Figure 3C, D, E). The results of the ratio of CD86^+^ and CD206^+^ expression confirmed this finding (Figure S9).

### Effects of M1M@Ds on induced ICD

Manganese ions can induce immunogenic cell death (ICD) in tumor cells, resulting in the release of damage-associated molecular patterns (DAMPs), such as the secretion of ATP, the externalization of CRT, and the liberation of HMGB1, thereby boosting the immunogenicity of the tumor microenvironment 26. As a result, we explored how M1M@D influenced induced ICD by analyzing the secretion of ATP, the externalization of CRT, and the liberation of HMGB1.

First, the cells in the groups with manganese ions secreted significantly more ATP into the cell culture medium than the cells in the groups without manganese ions. (Figure 3F). Subsequently, we employed CLSM and FCM to examine the externalization of CRT on B16F10 tumor cells. Under normal physiological conditions, CRT is localized within the endoplasmic reticulum lumen. However, during early apoptosis, the externalization of CRT is upregulated, and the protein is translocated to the cell membrane 26. The FCM results (Figure 3G) demonstrated that, in comparison to groups without manganese ions, the presence of manganese ions significantly increased CRT expression on the surface of B16F10 tumor cells. Figure 3H and S10 show that the membrane of B16F10 tumor cells in the groups with manganese ions exhibited obvious green fluorescence.

In typical physiological states, HMGB1 is primarily located within the nucleus. Nevertheless, during the initial phases of apoptosis, it moves from the nucleus into the cytoplasm 26. CLSM analysis confirmed the presence of HMGB1 release in the groups treated with manganese ions (Figure 3I, S11).

Given that hMnO_x_ also contributed to chemodynamic therapy, the reactive oxygen species (ROS) level in B16F10 tumor cells treated with M1M@Ds was detected by flow cytometry using a DCFH-DA fluorescent probe. The results demonstrated that the M1M@Ds group exhibited enhanced green fluorescence in B16F10 tumor cells in comparison to the control group (Figure S12), indicating that M1M@Ds induced substantial ROS accumulation, which consequently triggered oxidative stress injury.

These findings indicate that M1M@Ds can be internalized by B16F10 tumor cells and induce the polarization of M2 phenotype to the M1 phenotype. Furthermore, M1M@Ds also enhanced ICD in B16F10 tumor cells, as evidenced by increased ATP secretion, CRT exposure, and HMGB1 release, which are essential for initiating subsequent antitumor immune responses. Meanwhile, the DCFH-DA fluorescent probe assay by flow cytometry analysis indicated that M1M@Ds may play an important role in antitumor or immunomodulatory processes by promoting ROS production.

In summary, M1M@Ds demonstrated the multifunctional properties of regulating tumor microenvironment and enhancing antitumor immune response, which provided new ideas and possibilities for tumor immunotherapy. Through further research and development, M1M@Ds was expected to be an efficient and safe medicine for tumor therapy.

### Antitumor effects of M1M@Ds in a xenograft mouse model of B16F10 melanoma

**Figure 4:**
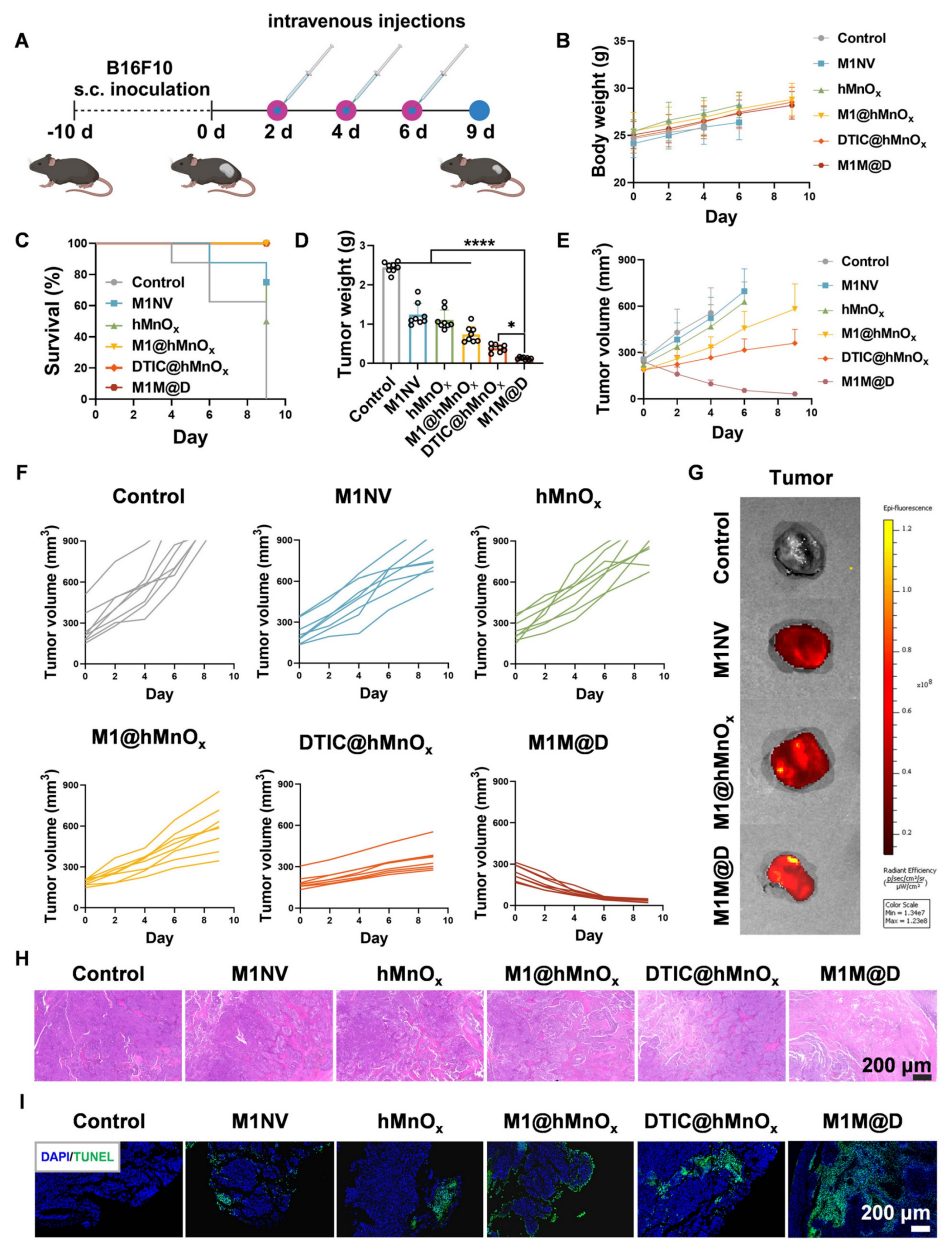
Antitumor effects of M1M@Ds in a xenograft mouse model of B16F10 melanoma.** (A)** Schematic diagram of the treatment protocol. **(B)** Changes in body weight of mice across different treatment groups. **(C)** Survival rates over time for the M1M@Ds group compared to the other groups. **(D)** Tumor weight for the tumors in each treatment group. **(E)** Summary of tumor growth curves and **(F)** the tumor growth curves for the tumors in different treatment group. Tumor growth monitoring for a treatment group was halted upon the death of one mouse in the group. Mice were euthanized if their tumor size exceeded 900 mm³. **(G)** *Ex vivo* fluorescence images of tumors 12 h after injection of M1NVs, M1@hMnO_x_s, and M1M@Ds labeled with Dir fluorescent dye.** ((H)** H&E and **(I)** TUNEL staining were performed to analyze tumor tissues from mice in different treatment group. The nuclei of the cells were labeled using DAPI, which is blue. All data are expressed as mean ± SD, *n* = 8. * p < 0.05, ** p < 0.01, *** p < 0.001, **** p < 0.0001.

Next, we assessed the *in vivo* antitumor activity of M1M@Ds using C57BL/6 mice with subcutaneous B16F10 melanoma tumors 27. Figure 4A illustrates the timeline of the animal experiments in the subcutaneous tumor model. Tumor samples were collected every two days following M1M@Ds injection over three consecutive treatments. After the administration of M1M@Ds in healthy rat, the concentration of DTIC in blood was measured at different time points (0.5 h, 1 h, 2 h, 4 h, 6 h, 8 h, 12 h,24 h, 48 h, 72 h, 96 h, 120 h) by UPLC-MS/MS (TSQ QUANTIVA, Thermo, US) (mobile phase A: 0.1% (*v/v*) formic acid aqueous solution, mobile phase B: acetonitrile) to calculate the drug half-life of M1M@Ds. The results showed that the drug half-life of M1M@Ds was 3.14 h (Figure S13).

The results showed that repeated dosing of M1M@Ds had no significant effect on the body weight of the mice relative to the control group (Figure 4B). Additionally, the survival duration of the mice treated with M1M@Ds was significantly extended (Figure 4C). Furthermore, the tumor weights in the M1M@Ds group were notably reduced compared to those in the other groups (Figure 4D). Also, based on the change in tumor size, we can see that M1M@Ds can effectively inhibit the tumor from growing (Figure 4E, F, S14, S15). M1M@Ds treatment leads to tumor regression from the first day of administration, significantly outperforming M1@hMnO_x_s and DTIC@hMnO_x_s. This immediate effect may result from the cytotoxic effect of DTIC, which can be exerted by targeting the tumor site with M1NV compared to DTIC@hMnO_x_s.

To demonstrate that the M1M@Ds can take advantage of the tumor site homing properties of the M1NVs for precise tumor targeting. We intravenously injected M1NVs, M1@hMnO_x_s, and M1M@D labeled with Dir fluorescent dye and collected mouse tumor tissues 12 h later and measured fluorescence signals. It can be seen that the tumor tissues in the M1NVs, M1@hMnO_x_s, and M1M@Ds groups have higher fluorescence signal intensity (Figure 4G), demonstration that M1M@Ds can be precisely targeted to melanoma sites *in vivo*. We also performed *in vivo* fluorescence imaging at the tumor site in mice within 48 hours of injection. The results showed that fluorescence appeared at the tumor site after 2 h and began to decay after 12 h. It also demonstrated that M1M@Ds could precisely target the melanoma site *in vivo* (Figure S16). We then examined the distribution of M1M@Ds in mice 48 hours after injection using fluorescence imaging. The results showed that M1M@Ds had the highest concentration in the liver, followed by the spleen and lung. The results indicated that M1M@Ds was mainly distributed in metabolic and excretory organs (Figure S17). In addition, we examined the distribution of M1M@Ds in organs and tumor tissues of mice 24 and 48 hours after injection using fluorescence imaging. The results again demonstrated that M1M@Ds can target tumor tissues (Figure S18).

Moreover, histological evaluations, such as hematoxylin and eosin (H&E) staining and terminal deoxynucleotidyl transferase (TdT)-mediated dUTP nick-end labeling (TUNEL) assays, demonstrated that M1M@Ds significantly decreased the density of cancer cells within tumor tissues (Figure 4H, I, S19). This observation was further corroborated by the reduced expression of Ki67 and Bcl2 in the subcutaneous tumor tissues of the M1M@Ds group (Figure S20, S21).

### Antitumor efficiency of M1M@Ds in lung metastatic tumor mode

Given the high metastatic potential of melanoma, we further explored the antitumor efficacy of M1M@Ds in a lung metastasis model. This model was created by injecting B16F10 tumor cells into the tail vein of C57BL/6 mice to evaluate the ability of M1M@Ds to suppress tumor cell metastasis.

Figure S22A illustrates the timeline of the animal experiments in the lung metastasis model. Lung tissue samples were collected every two days following three consecutive administrations of M1M@Ds. Repeated administration of M1M@Ds had no impact on the body weight of the mice when compared to the control group (Figure S22B). The results demonstrated that M1M@Ds significantly reduced the number and size of lung metastatic tumors (Figure S22C-E). H&E and TUNEL staining of lung tumor tissues revealed that M1M@Ds effectively induced apoptosis in tumor cells (Figure S22F-G, S23). Immunofluorescence analysis further indicated lower expression of Ki67 and Bcl2 in the M1M@Ds group compared to other groups (Figure S22H, S24). These findings suggest that M1M@Ds can effectively suppress tumor cell metastasis in the lungs.

In summary, M1M@Ds showed significant antitumor effects in the B16F10 melanoma xenograft mouse model and the B16F10 melanoma lung metastatic tumor model in C57BL/6 mice. By targeting tumor sites, inhibiting tumor cell proliferation and inducing their apoptosis, M1M@Ds was able to effectively reduce tumor growth and metastasis while prolonging the survival time of mice. Its high efficiency, targeting and safety made it an antitumor drug with potential clinical applications. While our results highlight promising therapeutic outcomes in melanoma models, we acknowledged limitations such as the need for long-term safety evaluations. Future work will explore the universality of this strategy across other cancer types and investigate scalable production methods.

In conclusion, M1M@Ds represented a potent immunochemotherapeutic strategy with translatable potential, bridging innate immunity and precision nanomedicine to combat immunosuppressive solid tumors.

### Effects of M1M@Ds on reprogramming tumor microenvironment *in vivo*

*In vitro* cellular experiments demonstrated that M1M@Ds induced the repolarization of M2 phenotype toward a M1 phenotype. TAMs in the tumor microenvironment are predominantly M2 TAMs, and repolarizing M2 macrophages to proinflammatory phenotype could improve the immunosuppressive state of the tumor microenvironment and synergistically enhance tumor immunotherapy. The antitumor efficacy of M1M@Ds treated tumors also suggests an antitumor immune response. Motivated by these results, we further explored the impact of M1M@Ds on reshaping the tumor microenvironment *in vivo*.

**Figure 5:**
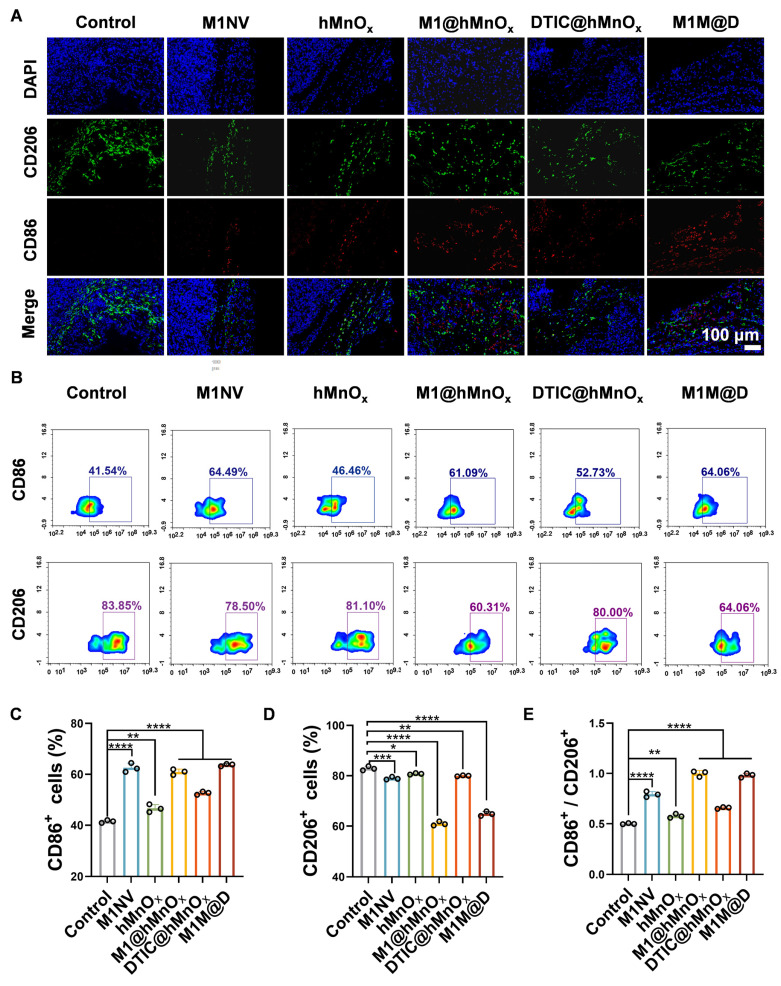
The M1M@Ds reprogramming tumor microenvironment *in vivo*. **(A)** the result of Fluorescence staining of CD86^+^ and CD206^+^ in the tumors. The nuclei of the cells were labeled using DAPI, which is blue.** (B)** The FCM results of CD86^+^ and CD206^+^ in the tumors. The quantifcation of the portion of **(C)** CD86^+^, **(D)** CD206^+^, and **(E)** the ratio of CD86^+^ and CD206^+^ subsets respectively. All data are expressed as mean ± SD, *n* = 3. day 9, the tumor tissues were collected for analysis. * p < 0.05, ** p < 0.01, *** p < 0.001, **** p < 0.0001.

Following the completion of the animal studies, mice were euthanized, tumors were collected, and immunofluorescence staining and flow cytometry were conducted to assess CD86^+^ and CD206^+^ expression levels. Immunofluorescence staining showed a notable rise in CD86^+^ expression and a reduction in CD206^+^ levels in tumor tissues treated with M1M@Ds (Figure 5A, S25). This conclusion was also supported by the results of the flow cytometry analysis (Figure 5B-E).

It has been reported in the literature that M1 TAMs can be polarized to release a variety of pro-inflammatory cytokines (TNF-α, IL-12, IL-23, IL-1β, and IL-6) to achieve their tumor-killing activity 28. In contrast, M2 macrophages, TGF-β, IL-1b, IL-4, IL-12, INF-γ, matrix metalloprotein (MMP), and vascular endothelial growth factor (VEGF) 29, have the ability to repair damaged tissue, stimulate angiogenesis, and promote tumorigenesis and progression 30. We evaluated the expression of inflammatory cytokines in tumor tissues. After treatment, the secretion levels of TNF-α, IFN-γ, IL-2, IL-6, and IL-12 were significantly increased, the secretion levels of IL-10 and TGF-β were significantly decreased (Figure S26). It indicated that M1M@Ds could promote the release of cytokines, induce macrophage polarization, and promote the occurrence and development of inflammatory responses.

In summary, we demonstrated that M1M@Ds effectively reprograms the TME through modulation of macrophage polarization. The M1NVs served as both a delivery vehicle and a bioactive component, which, together with hMnO_x_, skewing TAMs toward the antitumor M1 phenotype. This combinatorial approach not only enhanced DTIC's cytotoxicity but also reversed immunosuppressive TME signatures, as evidenced by increased CD86⁺ and reduced CD206⁺ cells.

### Impact of M1M@Ds on *in vivo* antitumor immune responses

*In vitro* cellular experiments demonstrated that M1M@Ds could promote immunogenic cell death (ICD), otentially triggering antitumor immune responses. The therapeutic outcomes of M1M@Ds also indicated the activation of antitumor immunity. Motivated by the results, we further investigated the role of M1M@Ds in modulating *in vivo* antitumor immune responses.

Results of the fluorescence staining showed increased expression of CRT and the cytoplasm of HMGB1 in tumors from the M1M@Ds treatment group compared to other groups (Figure S27, S28), suggesting the induction of ICD *in vivo*. In addition, the HMGB1-TLR4/AGER pathway is a central signaling axis connecting ICD and anti-tumor immune response. TLR4 and AGER, two of the most extensively studied receptors for HMGB1, have been demonstrated to mediate HMGB1 activity in a variety of immune cells through the activation of the NF-κB and IRF3 pathways, consequently inducing the production of pro-inflammatory cytokines and chemokines 31. The results indicated elevated expression of TLR4, AGER, IRF3, and NF-κB in tumor tissues (Figure S29), which suggested that M1M@Ds triggered alterations in the aforementioned pathway.

In addition, manganese ions can significantly promote the ability of host antigen-presenting cells, such as DCs and macrophages, to present tumor antigens through activation of the STING pathway, which promotes the infiltration of cytotoxic T-cells in tumor tissues and enhances the specific killing of tumor cells by these cells 23,24. We examined the expression of Sting, TBK1, IRF3, and NF-κB in tumor tissues (Figure S30), and found that M1M@Ds induced alterations in this pathway. Mature dendritic cells (DCs) typically express CD80/CD86 costimulatory molecules on their surface. By immunofluorescence staining, we observed a significant increase in the maturation of DCs in tumor-draining lymph nodes of mice in the M1M@Ds group (Figure S31). This suggests that M1M@Ds can promote the maturation of DCs, which in turn activates DAMPs, thereby initiating T cell-mediated immune responses.

**Figure 6:**
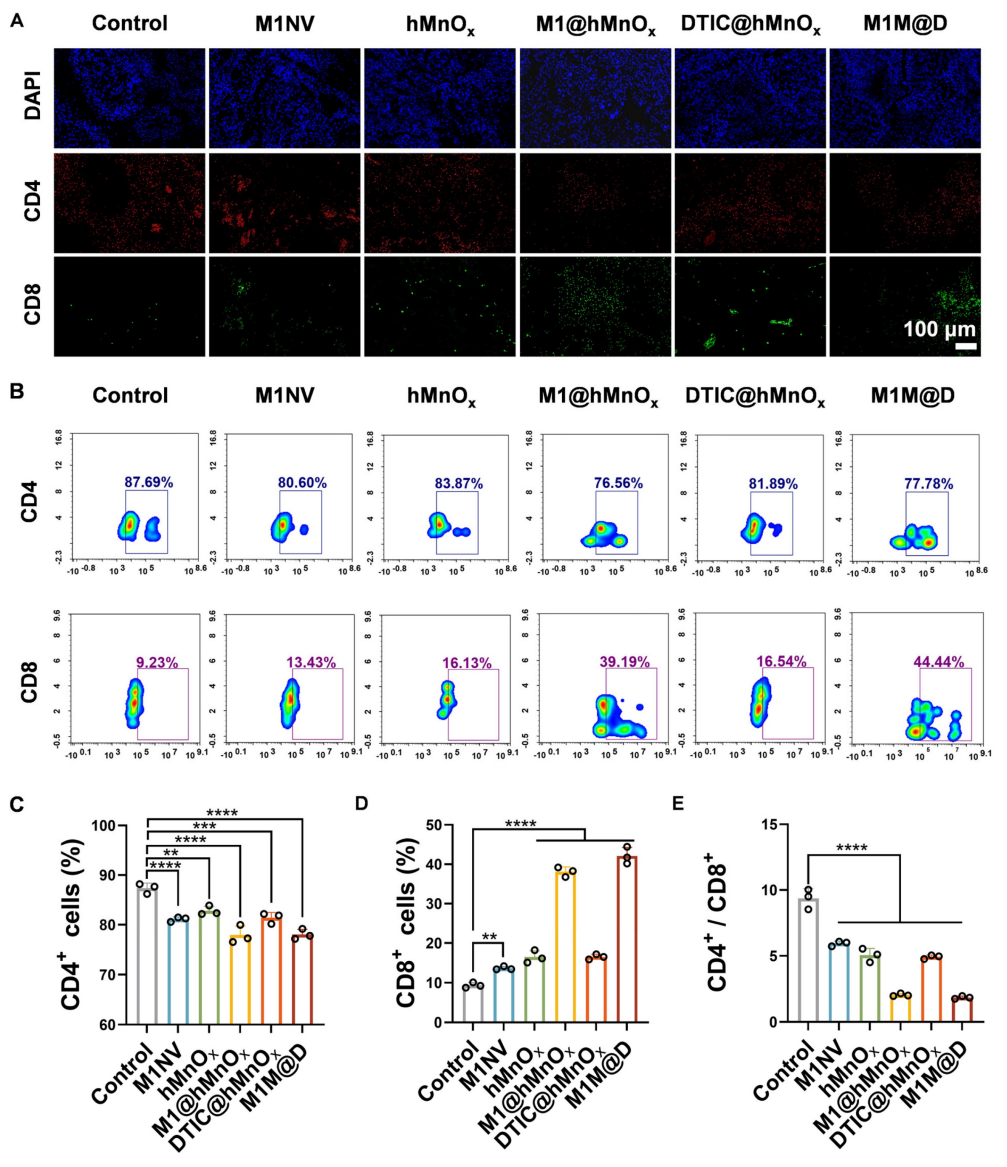
The M1M@Ds activated *in vivo* antitumor immune responses. **(A)** The results of fluorescence staining of CD4^+^ and CD8^+^ in the tumors. The nuclei of the cells were labeled using DAPI, which is blue. **(B)** The results of FCM of CD4^+^ and CD8^+^ in the tumors. The quantifcation of the portion of **(C)** CD4^+^, **(D)** CD8^+^, and **(E)** the ratio of CD4^+^ and CD8^+^ subsets respectively. All data are expressed as mean ± SD, *n* = 3. On day 9, the tumor tissues were collected for analysis. * p < 0.05, ** p < 0.01, *** p < 0.001, **** p < 0.0001.

In further experiments, tumors were collected on day 9 for immunofluorescence staining and flow cytometry to evaluate CD4^+^ and CD8^+^ expression levels. Immunofluorescence staining revealed a notable rise in CD8^+^ expression and a reduction in CD4^+^ content in tumor tissues from the M1M@Ds treatment group (Figure 6A, S32). This conclusion was also supported by the results of the flow cytometry analysis (Figure 6B-D, S35). Treatment with M1M@Ds also elevated the percentages of CD4^+^ and CD8^+^ T cells (Figure 6E). These findings confirmed the robust immune responses triggered by the M1M@Ds treatment. We also performed immunofluorescence staining analysis of lung metastases. The M1M@Ds group changed the proportion of CD4^+^, and CD8^+^ T cells compared to the other groups, further suggesting the activation of antitumor immunity (Figure S33, S34). To further explore the effect of M1M@D on systemic immune activation, we assessed the levels of CD4^+^ and CD8^+^ T cells in spleen tissues (Figure S36). The results of the flow cytometry revealed a notable rise in CD8 expression and a reduction in CD4 content in spleen tissues from the M1M@Ds treatment group. The ratio of CD4^+^ and CD8^+^ T cells changed in the M1M@Ds group compared to the other groups, indicating that systemic immunity was activated.

Our findings demonstrated that M1M@Ds effectively induces ICD in tumor cells, as evidenced by the release of DAMPs. These DAMPs serve as critical danger signals, promoting DC maturation and enhancing tumor antigen presentation. Consequently, this cascade activates robust T cell-mediated immune responses, including increased infiltration of cytotoxic CD8⁺ T cells. The combination of M1 macrophage-derived nanovesicles (M1NVs) and hMnO_x_ nanozymes not only potentiates ICD but also further amplifying the immunostimulatory effects. The chemotherapeutic agent DTIC contributes to tumor cell killing while synergizing with the immune-modulatory functions of M1M@Ds. Together, these mechanisms establish a self-reinforcing cycle of immune activation and tumor suppression.

However, further studies are needed to optimize dosing regimens, evaluate long-term immune memory effects, and assess potential off-target impacts. Future research should also explore the applicability of this strategy across diverse cancer types and in combination with other immunotherapies, such as checkpoint inhibitors.

In conclusion, M1M@Ds represented a promising approach to enhance antitumor immunity by integrating ICD induction, DC activation, and T cell priming, offering a potential pathway for more effective and durable cancer immunotherapy.

### *In vivo* safety evaluation of M1M@Ds

Lastly, healthy C57BL/6 mice were treated following the same protocols used in the antitumor efficacy studies to evaluate the biosafety of M1M@Ds.

**Figure 7:**
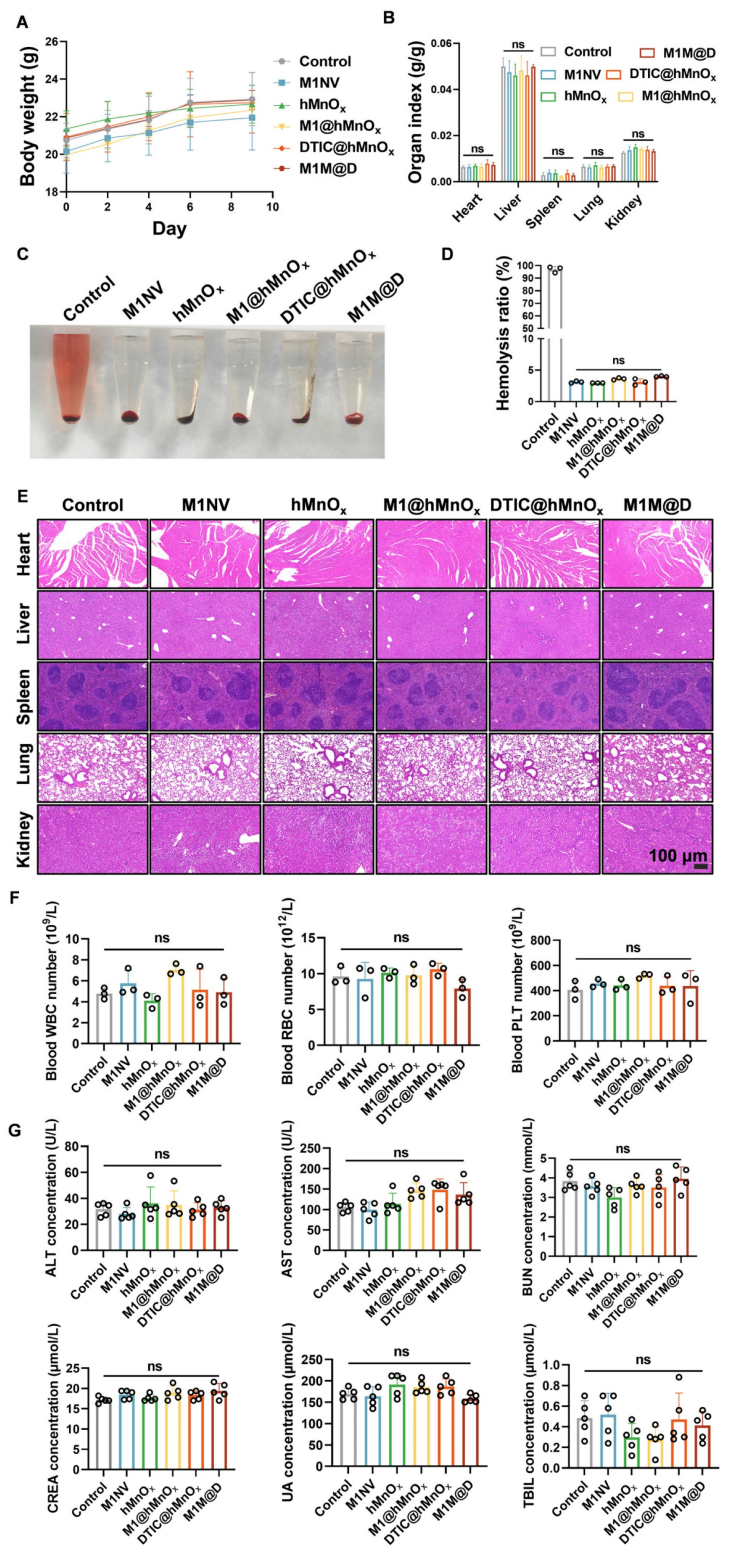
Safety evaluation of M1M@Ds *in vivo*. **(A)** The body weight and **(B)** organs index of mice after different treatments on day 9. (*n* = 5) **(C)** Photo and **(D)** Hemolysis ratio of mice after different treatments on day 9. **(E)** H&E staining of mice after different treatments on day 9. **(F)** The concentration of the white blood cells (WBC), the red blood cells (RBC), and the platelet (PLT) in blood of mice after different treatments on day 9. (*n* = 3) **(G)** The concentration of the alanine transaminase (ALT), the aspartate transaminase (AST), the blood urea nitrogen (BUN), the creatinine (CREA), the uric acid (UA), and the total bilirubin (TBIL) in serum of mice after different treatments on day 9. (*n* = 5), ns (not significant, p > 0.05).

As shown in Figure 7A and 7B, no significant changes in body weight or major organ indices (liver, kidney, spleen, lung, and heart) were observed among mice treated with M1NVs, hMnO_x_s, M1@hMnO_x_s, DTIC@hMnO_x_s, and M1M@Ds. We also evaluated whether the therapeutic dose induced a hemolytic reaction. Supernatants were detected by both visual observation and spectrophotometric assay. The results showed that the therapeutic dose did not cause any hemolytic reactions (Figure 7C, D). It also had a high safety profile. Major organs stained with H&E showed no discernible pathological alterations or inflammatory infiltration (Figure 7E). Additionally, red blood cell, white blood cell, and platelet counts, as well as liver and kidney function markers in serum biochemical tests, were all within normal ranges for mice treated with M1NVs, hMnO_x_s, M1@hMnO_x_s, DTIC@hMnO_x_s, and M1M@Ds, showing no significant differences compared to the control group (Figure 7F, G).

To investigate whether manganese ions in M1M@Ds would trigger neurotoxicity *in vivo*, we used ICP-MS (Agilent 7800) to quantify the manganese level in the brain tissue of mice. The results showed that there was no significant difference in the manganese levels between the treatment group and the control group, indicating that manganese ions in M1M@Ds were difficult to cross the blood-brain barrier and did not accumulate in the brain (Figure S37). H&E staining of brain tissue showed that no obvious neuronal degeneration, necrosis or inflammatory infiltration was observed in the treatment group (Figure S38). The TUNEL staining further confirmed that manganese ion exposure did not cause significant neuronal apoptosis (Figure S39). In conclusion, manganese ions in M1M@Ds did not exhibit significant neurotoxicity at therapeutic doses, and its safety was verified.

In conclusion, M1M@Ds showed good biocompatibility and low toxicity in safety evaluation, which provides a solid foundation for its clinical translation. Its unique three-layer structural design and the selection of biological components may be key factors for its safety. Future studies should further validate its long-term safety and quality control for large-scale production to promote its application in the clinic.

## Conclusions

In this study, we successfully developed an innovative nanozyme-boosted biomimetic macrophage-derived TME reshaping nanoplatform, termed M1M@D, which integrates M1 macrophage-derived NVs (M1NVs), hollow virus-spiky manganese oxide (hMnO_x_) nanozymes, and the chemotherapeutic agent dacarbazine (DTIC). The nanoplatform exhibits a unique three-layer structure, enabling precise tumor targeting, localized drug release, and effective tumor cell killing. Moreover, M1M@D exhibits a significant capacity to reprogram tumor-associated macrophages (TAMs) from the immunosuppressive M2 state to the pro-inflammatory M1 state, effectively altering the immunosuppressive TME. Furthermore, the hMnO_x_ nanozymes boost ICD in tumor cells, stimulate DC maturation, and enhance T-cell-driven antitumor immune responses. With its straightforward fabrication process, high production yield, and scalability, M1M@D represents a promising and versatile platform for future translational research and clinical applications in cancer immunotherapy, offering a dual functionality in precision drug delivery and immunomodulation.

## Supplementary Material

Supplementary methods and figures.
