# Supplementary material for: Synergistic tumor microenvironment modulation enabled by a nanozyme-boosted biomimetic macrophage-derived nanovesicle for highly efficient antitumor therapy
Source: Theranostics. 2025 Jul 11;15(16):7925–39. doi: 10.7150/thno.114467 (PMC12374549; doi:10.7150/thno.114467)
Supplement: Supplementary file 1 — Supplementary methods and figures. [file thnov15p7925s1.pdf]

Supporting Information

Synergistic tumor microenvironment modulation enabled by a nanozyme-boosted biomimetic macrophage-derived nanovesicle for highly efficient antitumor therapy

Yue Su [a, b, §], Haibin Wu [c, §], Ke Duan [c, §], Jiahao Xie [b], Weitao Huang [a, d], Xiaozhou Mou [b], Xiangming Ye [a], Yeyu Shen [d], Ting Li [b], Junjia He [d], Luoqin Fu [b], Yin Wang [e], Liping Wen [f, *], Qiong Bian [a, d, *], Mingang Zhu [e, *], Xiangmin Tong [g, *]

[a] Center for Rehabilitation Medicine, Rehabilitation and Sports Medicine Research Institute of Zhejiang Province, Department of Rehabilitation Medicine, Zhejiang Provincial People's Hospital, Affiliated People's Hospital, Hangzhou Medical College, Hangzhou, Zhejiang 310014, China.

[b] Clinical Research Institute, Zhejiang Provincial People’s Hospital, Affiliated People’s Hospital, Hangzhou Medical College, Hangzhou, Zhejiang 310014, China.

[c] School of Pharmaceutical Sciences, Hangzhou Medical College, Hangzhou 311399, Zhejiang, China

[d] Center for Plastic & Reconstructive Surgery, Department of Dermatology, Zhejiang Provincial People’s Hospital, Affiliated People’s Hospital, Hangzhou Medical College, Hangzhou, Zhejiang 310014, China.

[e] Department of Dermatology, the First People’s Hospital of Jiashan, Jiashan Hospital Affiliated to Jiaxing University, Jiaxing 314100, Zhejiang, China.

[f] Department of Urology, the First People's Hospital of Fuyang District, Hangzhou, Zhejiang 311400, China

[g] Department of Hematology, the Affiliated Hangzhou First People’s Hospital, Westlake University School of Medicine, Hangzhou, 310006, China

Corresponding Author

*Email: tongxiangmin@163.com (Xiangmin Tong)

*Email: bianqiong@zju.edu.cn (Qiong Bian)

*Email: zmg225@163.com(Mingang Zhu)

*Email: winleopard@163.com (Liping Wen)

[§]These authors contributed equally to this work.

METHODS

Materials. DTIC was obtained from Aladdin (Shanghai, China). Recombinant Mouse M-CSF was purchased from Amizona (Hangzhou, China). Recombinant Murine IFN-γ was obtained from Peprotech (Wuhan, China). The calreticulin Polyclonal antibody was purchased from proteintech (Shanghai, China). The HMGB1 Rabbit mAb was purchased from ABclonal (Shanghai, China). The ATP Assay Kit was purchased from Beyotime Biotechnology (Shanghai, China). The Ficoll-Plaque PLUS (density: 1.077 ± 0.001 g/ml) was purchased from Cytiva (Shanghai, China). The APC Anti-Mouse CD86, PE Anti-Mouse CD206, Brilliant Violet 421 Anti-Mouse CD4, and PE Anti-Mouse CD8a antibodies were sourced from Biolegend (San Diego, CA, USA). The Mouse TNF-α ELISA Kit, the Mouse IFN-γ ELISA Kit, the Mouse IL-2 ELISA Kit, the Mouse IL-6 ELISA Kit, the Mouse IL-12 ELISA Kit, the Mouse IL-10 ELISA Kit, the Mouse TGF-β ELISA Kit, the Mouse TLR4 ELISA Kit, the Mouse AGER ELISA Kit, the Mouse IRF3 ELISA Kit, the Mouse NF-κB ELISA Kit, the Mouse Sting ELISA Kit, the Mouse TBK1 ELISA Kit were sourced from Jonln Bio (Shanghai, China). All other chemicals and solvents were acquired from Aladdin (Shanghai, China).

Cell culture. Mouse B16F10 tumor cells were maintained in Dulbecco`s Modified Eagle`s Medium (DMEM) containing 10% fetal bovine serum (FBS), penicillin (100 units/mL), and streptomycin (100 μg/mL). The cells were incubated at 37°C in a humidified environment with 5% CO_2.

Animals. The male C57BL/6 mice (6-8 weeks old) were purchased from Shanghai Qizhen Laboratory Animal Co. Ltd. (Shanghai, China). All procedures performed were in accordance with Zhejiang Provincial People`s Hospital`s guidelines for the welfare of experimental animals. The study was approved by the animal ethics committee of Zhejiang Provincial People`s Hospital.

Fabrication, morphological analysis and particle size measurement of M1M@Ds. Primary macrophages were induced into stable M1 macrophages after extraction from mouse bone marrow. M1 macrophages were centrifuged, resuspended in PBS, and processed through polycarbonate membranes with pore sizes of 1 μm, 400 nm, and 200 nm using an extrusion device to generate nanoscale vesicular M1NVs. M1NVs were purified from cellular debris by centrifugation at 4℃ for 20 minutes at 4,000 ×g then 10,000 ×g. The supernatant was then withdrawn and ultracentrifuged at 100,000 ×g for 1 hour at 4℃. The subsequent pellet was washed five times with PBS and

resuspended in PBS. Next, the $hMnO_x$s was synthesized according to a previous literature report [1]. In a typical synthesis procedure, $KMnO_4$ and SSNs were dissolved in deionized water, respectively. Next, 30 mL of $KMnO_4$ solution (10 mg/mL) was slowly added to 4 mL of SSNs solution (10 mg/mL) under ultrasonication. After 6 h of ultrasonication, the SSN@MnO_xs was obtained by centrifugation and washed for several times with ethanol and deionized water. To remove the core template SSNs, the as-prepared SSN@MnO_xs was dissolved in aqueous solution of Na_2CO_3, and the resulting mixture was stirred for 12 h at 60 °C. Afterward, the brown-black $hMnO_x$s was collected by centrifugation, washed several times with deionized water and ethanol to remove the remaining reactants. Next, 5 mg of DTIC was weighed precisely and dissolved into 1 mL of aqueous methanol solution to form a 5 mg/mL aqueous methanol solution of DTIC. Subsequently, 800 μL of DTIC methanol solution (5 mg/mL) was mixed with 500 μL of $hMnO_x$s (10 mg/mL) and stirred at room temperature for 24 h to produce DTIC@$hMnO_x$s. M1M@Ds were then obtained by passing M1M@Ds through polycarbonate membranes with pore diameters of 400 nm using an extruder at a ratio of M1NVs:DTIC@$hMnO_x$s = 100:1. Free M1NVs was removed by density gradient centrifugation (30%/60% sucrose, 100,000 ×g /4 h). When particles/molecules are physically extruded, the applied pressure forces the identically charged particles to break through the electrostatic repulsion barrier and enter the short-range range of action (typically <2 nm) where van der Waals forces or hydrophobic interactions dominate. Our physical extrusion can force the negatively charged M1NVs and DTIC@$hMnO_x$s to temporarily approach each other to form a stable structure. When DTIC@$hMnO_x$s is wrapped into M1NV, the positive charge inside M1NV and negatively charged DTIC@$hMnO_x$s will form a stable concentric circle structure. The hydrodynamic sizes of M1M@Ds were determined using a Malvern Nano-ZS90 instrument (Malvern Instruments, Malvern, UK) at 25°C. Freshly prepared nanoparticles were dispersed in water for measurement. The results are reported as average sizes ± standard deviations based on three independent measurements.

Cellular uptake was evaluated using confocal laser scanning microscopy (CLSM) and flow cytometry (FCM). The cellular uptake of M1NVs, M1@$hMnO_x$s, and M1M@Ds at equivalent M1 macrophage concentrations was visualized using CLSM and quantified via FCM. Briefly, labeled M1NVs, M1@$hMnO_x$s, and M1M@Ds with the fluorescent dye Dio. B16F10 tumor

cells were seeded in glass-bottom dishes at a density of 3×10^4 cells/well. After a 24 h incubation to allow cells to adhere, 100 μL of fresh medium containing M1NVs, M1@hMnO$_x$s, and M1M@Ds were added and incubated for 0, 2, 6, 8, 12, and 24 h. Subsequently, the supernatant was removed and the cells were counterstained with Hoechst for CLSM observations (Leica DMI8 laser confocal microscope). Additionally, for the flow cytometry analysis, after incubation with M1NVs, M1@hMnO$_x$s, and M1M@Ds, the cells were digested and washed with PBS and further centrifuged at 1200 rpm for 5 min. After three cycles of centrifugation and resuspension, the intracellular fluorescence of Dio was detected using FCM (NovoCyte Advanteon, Agilent).

Extracellular ATP release assays, CRT exposure, and HMGB1 secretion. To measure ATP secretion, B16F10 tumor cells were exposed to M1NVs, hMnO$_x$s, M1@hMnO$_x$s, and M1M@Ds for 24 hours. The culture medium was collected, and dead cells were eliminated via centrifugation. The supernatants were used to quantify ATP levels using an ATP assay kit (Beyotime). The cells treated with M1NVs was as controls for all the three assays. CRT exposure of B16F10 tumor cells induced by M1NVs, hMnO$_x$s, M1@hMnO$_x$s, and M1M@Ds was evaluated by the CLSM and FCM. Briefly, 3×10^4 B16F10 tumor cells were plated in glass-bottom dishes and treated with M1NVs, hMnO$_x$s, M1@hMnO$_x$s, and M1M@Ds for 24 hours. After incubation at 37°C, the cells were washed with PBS (pH 7.2) and incubated with a calreticulin polyclonal antibody for 12 hours. Then cells were washed with phosphate buffered saline (PBS, pH = 7.2), incubated with Rabbit IgG FITC for 1 h. Then cells were washed with phosphate buffered saline (PBS, pH = 7.2), stained with 4',6-diamidino-2-phenylindole (DAPI), and then observed under the CLSM. As for the FCM analysis, after an incubation with M1NVs, hMnO$_x$s, M1@hMnO$_x$s, and M1M@Ds. Then the cells were washed with PBS and incubated with the calreticulin Polyclonal antibody for 12 h. Then cells were washed with phosphate buffered saline (PBS, pH = 7.2), incubated with Rabbit IgG FITC for 1 h. Then cells were washed with phosphate buffered saline (PBS, pH = 7.2), stained with 4',6-diamidino-2-phenylindole (DAPI), analyzed by a flow cytometry system (NovoCyte Advanteon, Agilent). To determine the release of HMGB1 by B16F10 tumor cells upon M1M@Ds treatment, the cells were treated with M1NVs, hMnO$_x$s, M1@hMnO$_x$s, and M1M@Ds. for 24 h. After following incubation for 24 h, the cells were washed and incubated with the HMGB1 Rabbit mAb for 12 h. Then cells were washed with phosphate buffered saline (PBS, pH = 7.2), incubated with Rabbit IgG FITC for 1 h. Then cells were washed with phosphate buffered saline (PBS, pH = 7.2),

stained withDAPI, and then observed under the CLSM.

Development of the B16F10 Melanoma Xenograft Model and Assessment of the Antitumor Activity of M1M@Ds. The protocol research was reviewed and approved by the Animal Ethics Committee of Zhejiang Provincial People's Hospital. To establish the xenograft mouse model, B16F10 tumor cells (3×10^6) were inoculated into the right flanks of C57BL/6 mice. One week later, tumor-bearing mice were divided into six groups (n = 8 for each group), namely a Control group, a M1NVs group, a $hMnO_x$s group, a M1@$hMnO_x$s group, a DTIC@$hMnO_x$s group, and a M1M@Ds group. For the M1M@Ds group, mice were treated with 200 μL of M1M@Ds every two days for 3 times. The tumor volume and body weight of the mice were measured and recorded. The tumor volume was determined using the formula: tumor volume (mm^3) = $width^2$ (mm^2) × length (mm) × 0.5. After 9 d, the mice were sacrificed, and the tumors were weighed. Animals were euthanized when they showed signs of imperfect health or when the size of tumors exceeded 900 mm^3. In addition, the other eight of mice in M1M@Ds group were selected to evaluate the survival time within 9 days.

Establishment of the B16F10 lung metastatic tumor mode and Assessment of the Antitumor Activity of M1M@Ds. The study was approved by the animal ethics committee of Zhejiang Provincial People`s Hospital. To establish lung metastatic tumor mode, B16F10 tumor cells (1×10^6) were injected into C57BL/6 mice via tail vein. One week later, tumor-bearing mice were divided into six groups (n = 5 for each group), namely a Control group, a M1NVs group, a $hMnO_x$s group, a M1@$hMnO_x$s group, a DTIC@$hMnO_x$s group, and a M1M@Ds group. For the M1M@Ds group, mice were treated with 200 μL of M1M@Ds every two days for 3 times. The body weight of mice was recorded. After 9 d, the mice were sacrificed, mouse lung tissue was then collected and photographed. Metastatic nodules in the lungs were counted and measured under a stereomicroscope. In addition, lung tissues were fixed and embedded in paraffin, and then subjected to H&E and immunofluorescence staining to assess histopathology. In addition, the other five of mice in M1M@Ds group were selected to evaluate the survival time within 9 days.

***In Vivo* safety evaluation.** To evaluate the in vivo toxicity of M1M@Ds, healthy C57BL/6 mice (aged 6-8 weeks) were used. The mice were divided into six groups (n = 5 per group). M1M@Ds were administered to the mice on days 2, 4, and 6. The experimental design, including the grouping and other parameters, was identical to that used in the in vivo antitumor efficacy studies.

The body weights of the mice were monitored daily until day 9. On day 9, blood samples were collected for analysis of blood cell counts and biochemical parameters. The biochemical parameters measured included serum the alanine transaminase (ALT), the aspartate transaminase (AST), the total bilirubin (TBIL), the blood urea nitrogen (BUN), the uric acid (UA), and the creatinine (CREA), which were used to assess potential hepatic and renal toxicity. Brain tissue was taken and ground into powder and passed through a 200 mesh sieve, then the sample was put into a sample bottle, and an appropriate amount of diluent (e.g., nitric acid or sodium hydroxide solution) was added and stirred well. The treated samples were passed through an Inductively coupled plasma-mass spectrometry (ICP-MS, Agilent 7800) to collect the ion signal data of the samples, and finally the elemental concentration was calculated by a calibration curve to evaluate whether manganese ions can cause neurotoxicity *in vivo*.

Flow cytometry. For immunocyte infiltration in tumors: The tumors were collected on day 9 after different treatments, cut into small pieces, and homogenized to form a single cell suspension. Then the Ficoll-Plaque PLUS was used to extract the lymphocytes. For macrophage polarization and T cells infiltration tests: The DCs were stained with APC/Cy7 anti-CD11c (Biolegend), BV421-F4/80 (Biolegend), APC anti-CD86 (Biolegend), PE anti-CD206(Biolegend), FITC anti-CD3 (Biolegend), BV421 anti-CD4 (Biolegend) and PE anti-CD8a (Biolegend). For T cell infiltration in spleen, the cells were stained with FITC anti-CD3 (Biolegend), BV421 anti-CD4 (Biolegend) and PE anti-CD8a (Biolegend). The stained cells were measured and analyzed on a flow cytometer (NovoCyte Advanteon, Agilent).

Statistical analysis. Data are expressed as mean ± standard deviation (SD). Statistical significance was determined using one-way ANOVA with Tukey's multiple comparisons test. Significance thresholds were set as: ns (not significant, $p > 0.05$), *$p < 0.05$, **$p < 0.01$, ***$p < 0.001$, and ****$p < 0.0001$. All analyses were performed using Prism software (version 9.0, GraphPad Software).

1. Yang G, Xu L, Chao Y, et al. Hollow MnO_2 as a tumor-microenvironment-responsive biodegradable nano-platform for combination therapy favoring antitumor immune responses. Nat Commun. 2017; 8: 902.

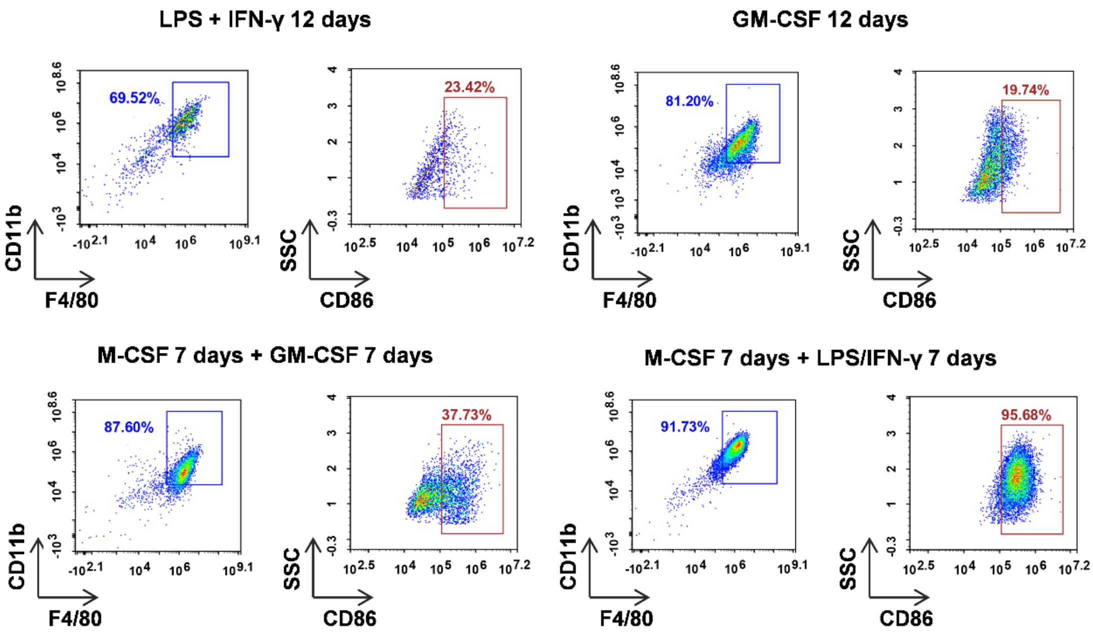

Figure S1. The process of trying the induction protocols of M1 macrophages.

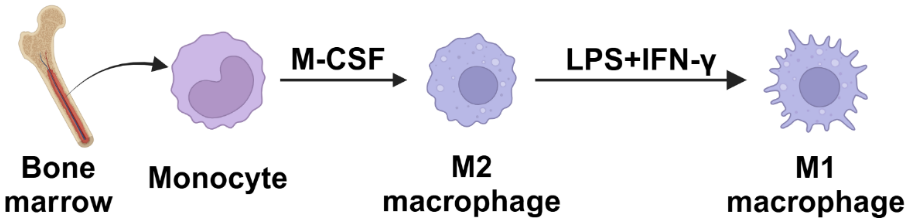

Figure S2. Induction of M1 macrophages.

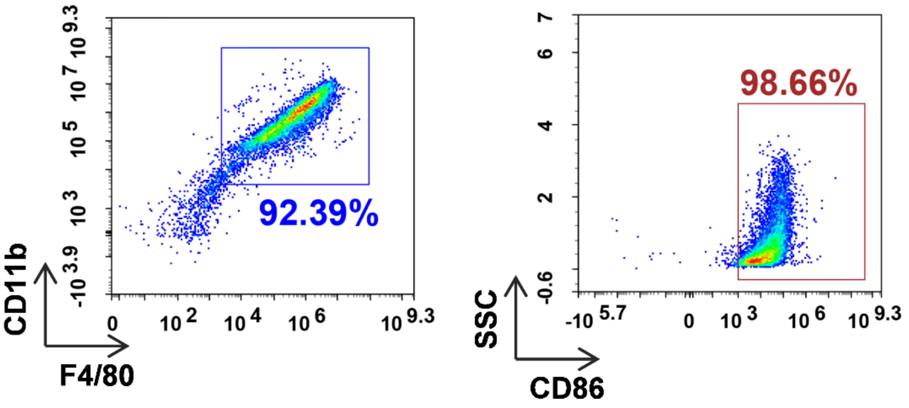

Figure S3. Flow cytometry of M1 macrophages for detection of F4/80, CD11b, and CD86.

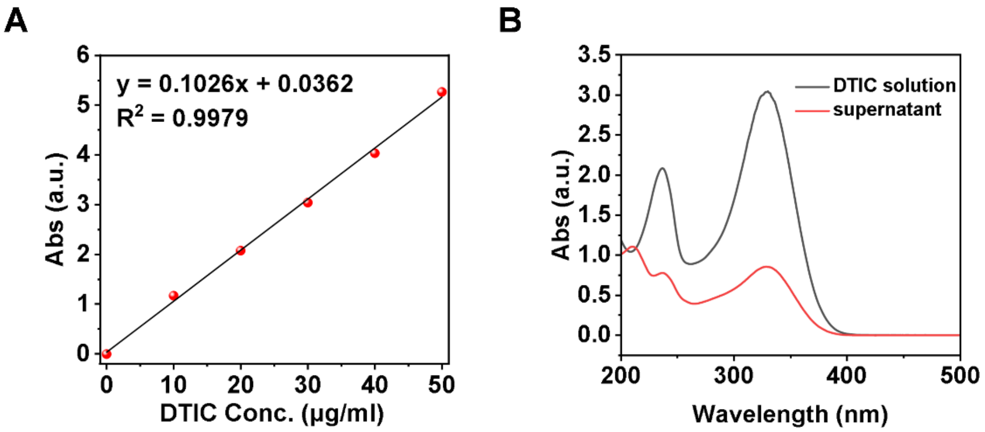

Figure S4. The loading rate of DTIC@hMnO$_x$s was measured by UV spectrophotometer. (A) Standard curve for DTIC solution obtained at the wavelength of 328 nm. (B) The UV-vis spectra of the equally diluted initial DTIC solution (30 μg/mL) and centrifuged supernatant after loading into hMnO$_x$s.

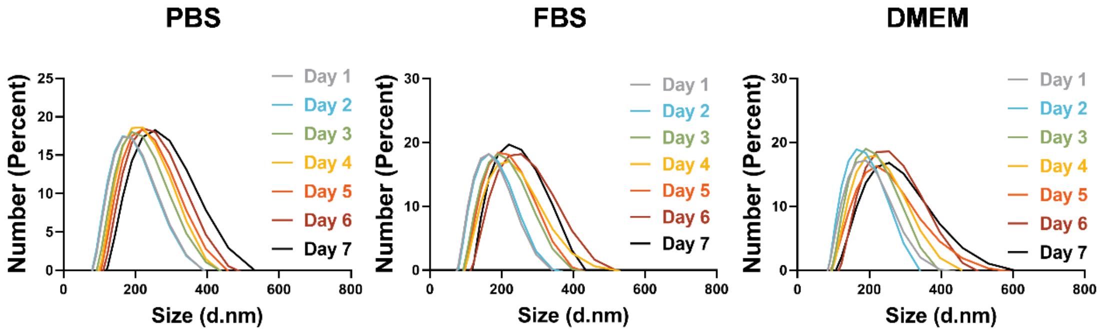

Figure S5. Particle size distribution of M1M@Ds in PBS, FBS and DMEM measured using DLS for 7 days.

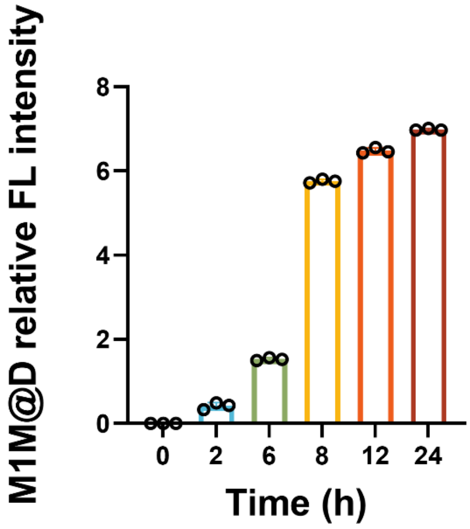

Figure S6. Relative FL intensity results of the cellular uptake of M1M@Ds.

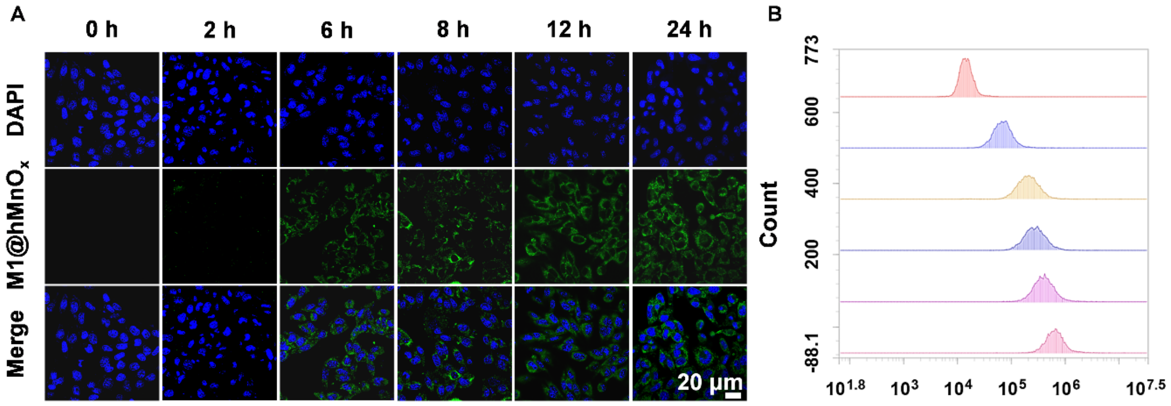

Figure S7. Cellular uptake of M1NVs. (A) Observations based on confocal microscopy and (B) FCM evaluation of the cellular uptake of M1NVs.

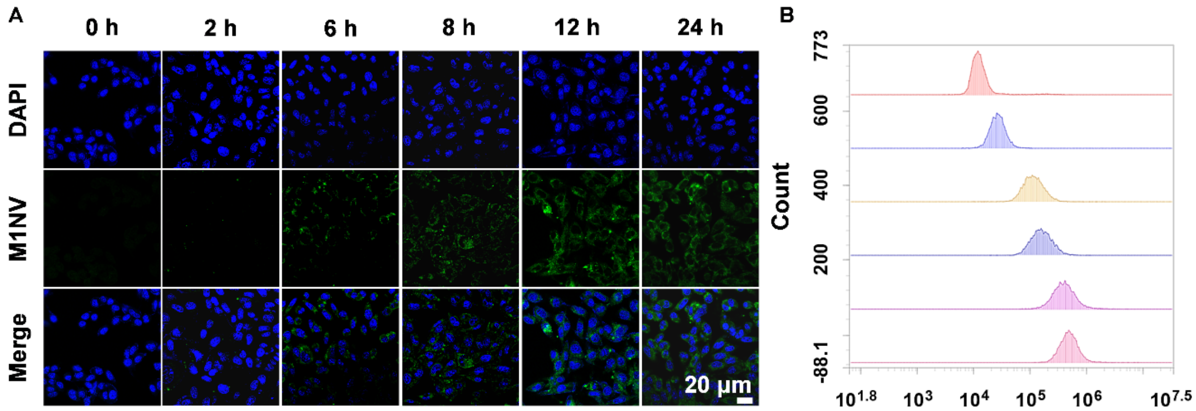

Figure S8. Cellular uptake of M1@hMnO$_x$s. (A) Observations based on confocal microscopy and (B) FCM evaluation of the cellular uptake of M1@hMnO$_x$s.

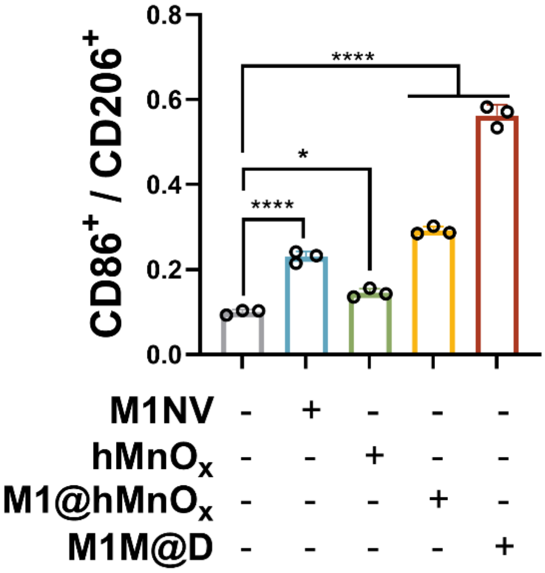

Figure S9. Ratio of $CD86^+$ and $CD206^+$ expression in Control, M1NVs, $hMnO_x$s, M1NV@$hMnO_x$s, and M1M@Ds group. Data are presented as mean ± SD, n = 3. * $p < 0.05$, ** $p < 0.01$, *** $p < 0.001$, **** $p < 0.0001$.

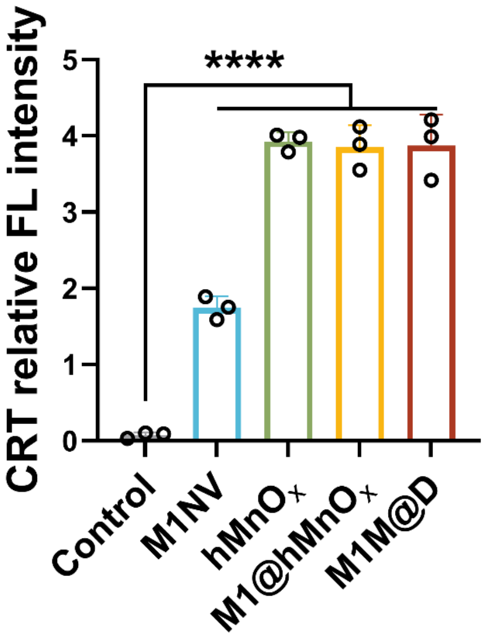

Figure S10. Relative FL intensity results of CRT. Data are presented as mean ± SD, $n = 3$. ns, non-significant ($p > 0.05$), * $p < 0.05$, ** $p < 0.01$, *** $p < 0.001$, **** $p < 0.0001$.

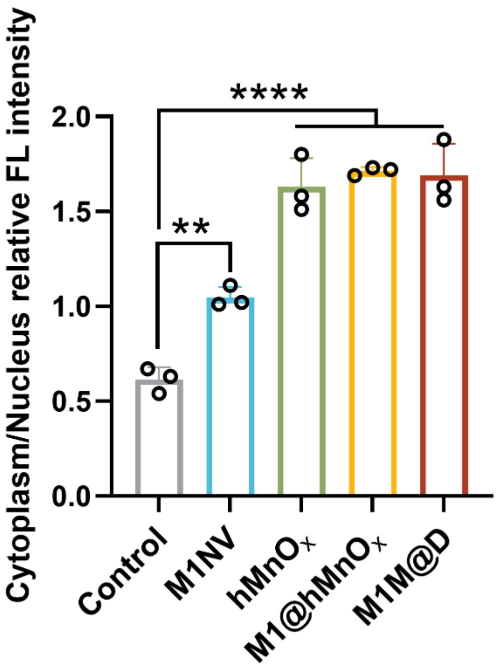

Figure S11. Relative HMGB1 FL intensity results of Cytoplasm/Nucleus. Data are presented as mean ± SD, $n = 3$. ns, non-significant ($p > 0.05$), * $p < 0.05$, ** $p < 0.01$, *** $p < 0.001$, **** $p < 0.0001$.

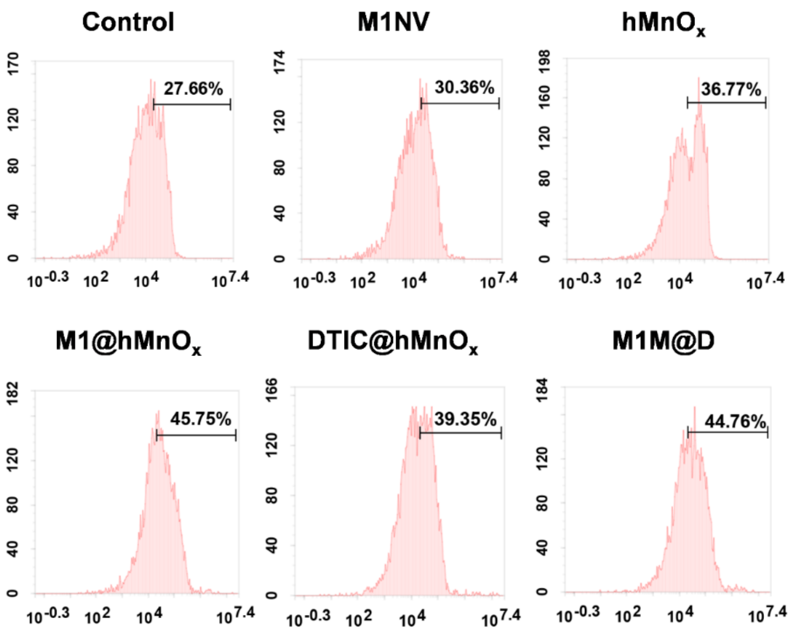

Figure S12. Expression of ROS green fluorescence in tumor cells after 24 hours of different treatments detected by flow cytometry.

PK parameter	M1M@Ds
$t_{1/2}$ (h)	3.14 ± 0.15
C_{max} (μg/mL)	1.366 ± 0.299
$AUC_{(0\text{-}inf)}$ (μg·h/mL)	10.761 ± 3.099

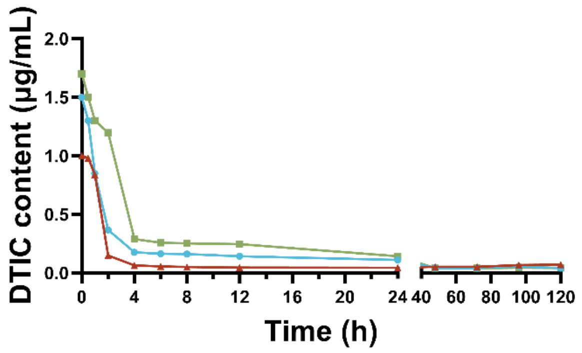

Figure S13. *In vivo* pharmacokinetic analysis, UPLC-MS/MS to determine the drug-time profile after a single tail vein injection of M1M@Ds in SD rats, n=3. Each profile represents data from one rat.

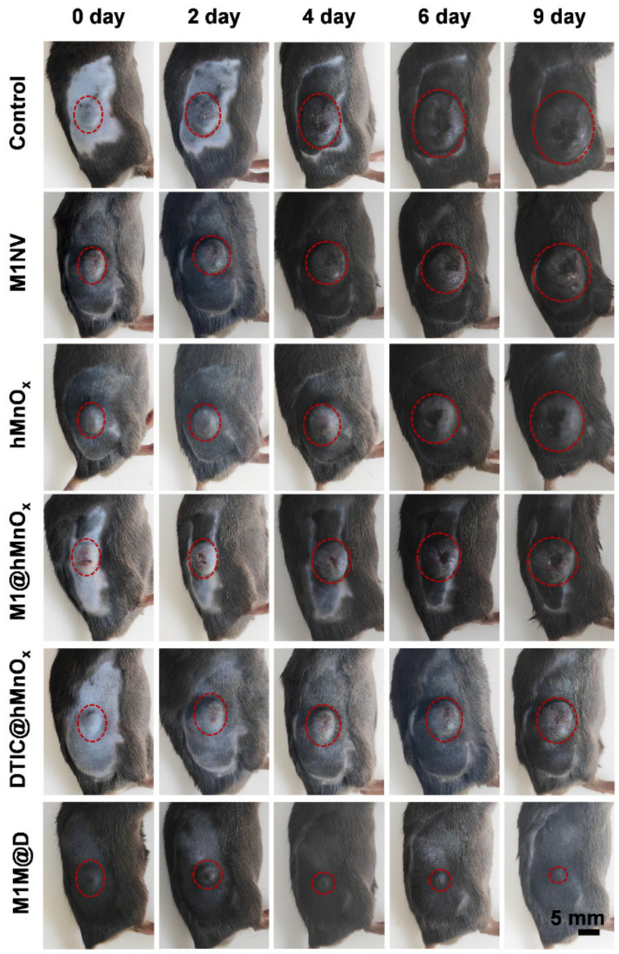

Figure S14. The tumor size changes in live mice.

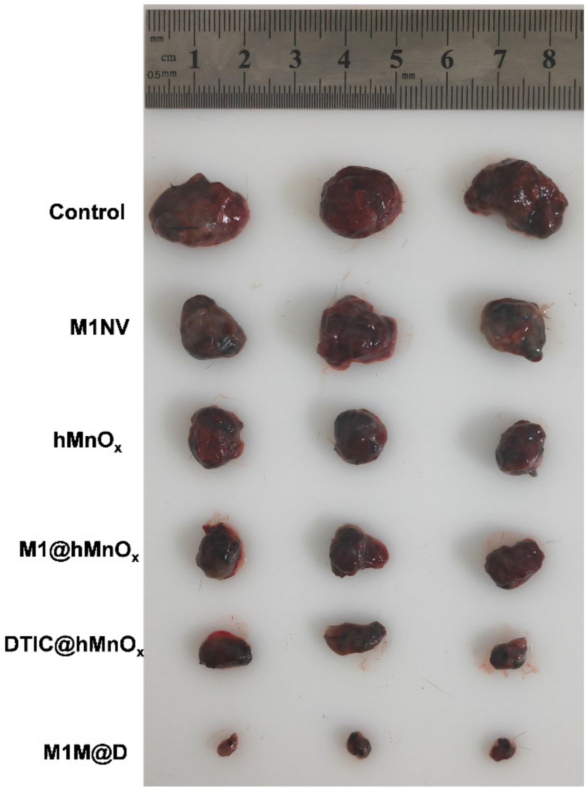

Figure S15. The size of tumors in mice at the experimental endpoint.

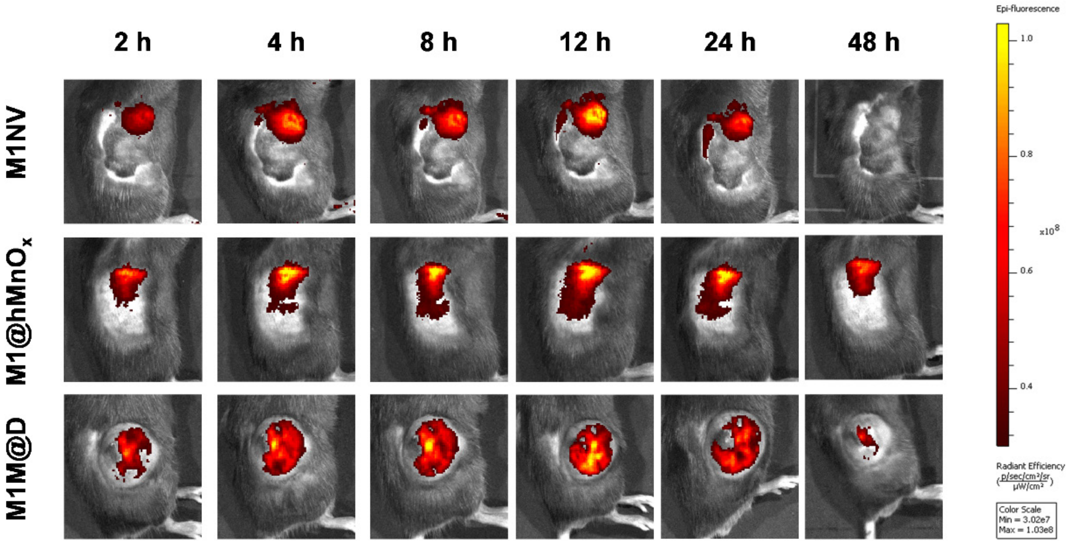

Figure S16. *In vivo* fluorescence imaging of M1NVs, M1@hMnO$_x$s, and M1M@Ds in the mouse tumors within 48 h after the injection.

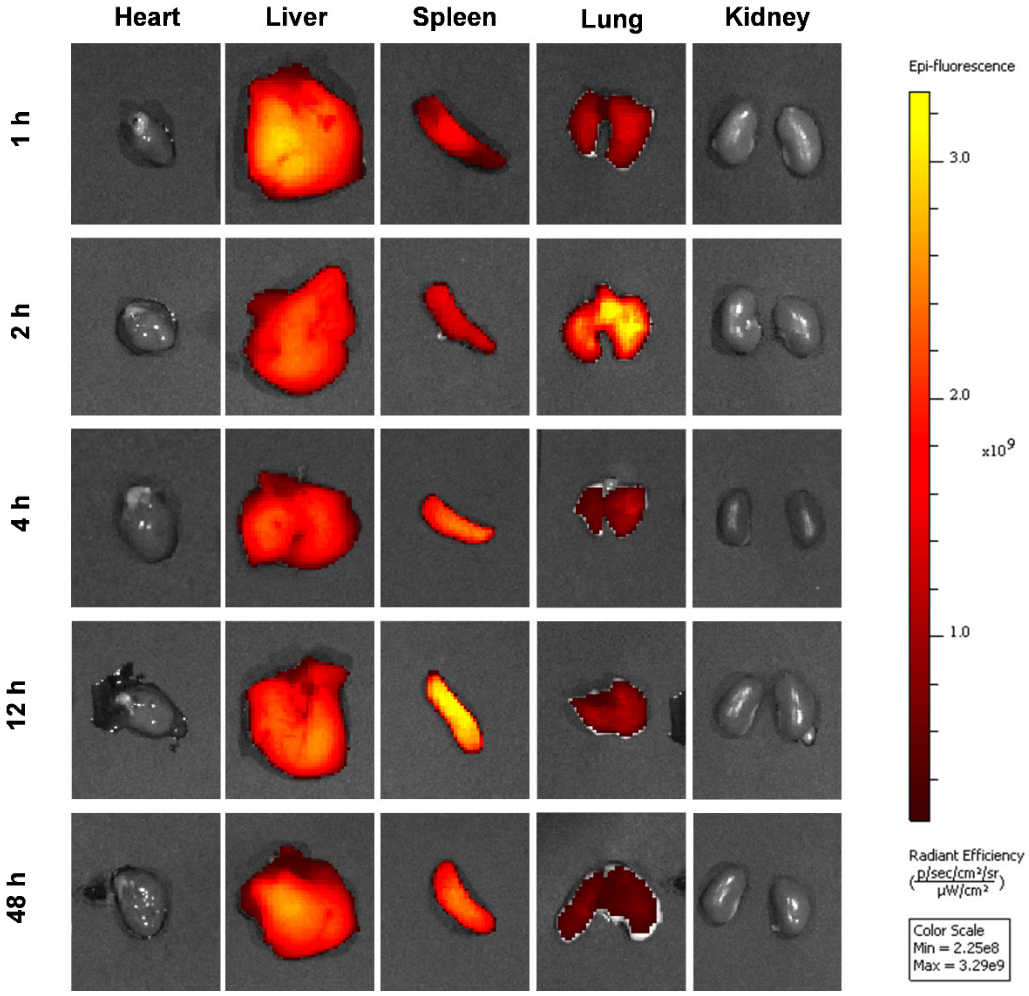

Figure S17. Fluorescence imaging of the distribution of M1M@Ds in mice within 48 h after injection.

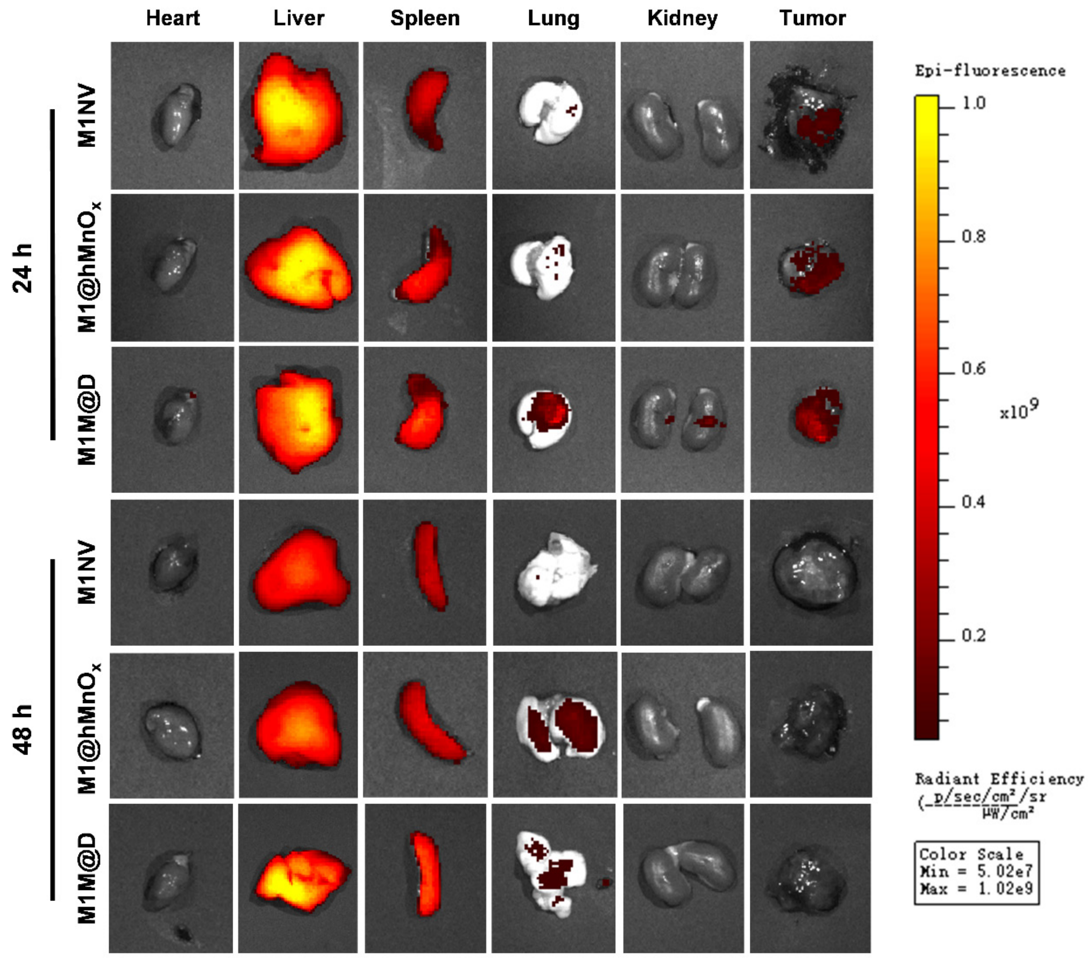

Figure S18. Fluorescence imaging of the distribution of M1NVs, M1@hMnO$_x$s, and M1M@Ds in mice 24 h and 48 h after injection.

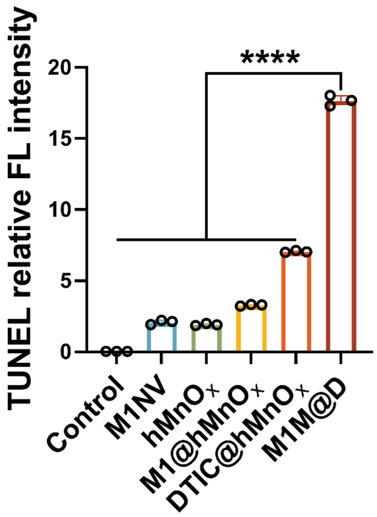

Figure S19. Relative FL intensity results of TUNEL in tumor tissues. Data are presented as mean ± SD, $n = 3$, **** $p < 0.0001$.

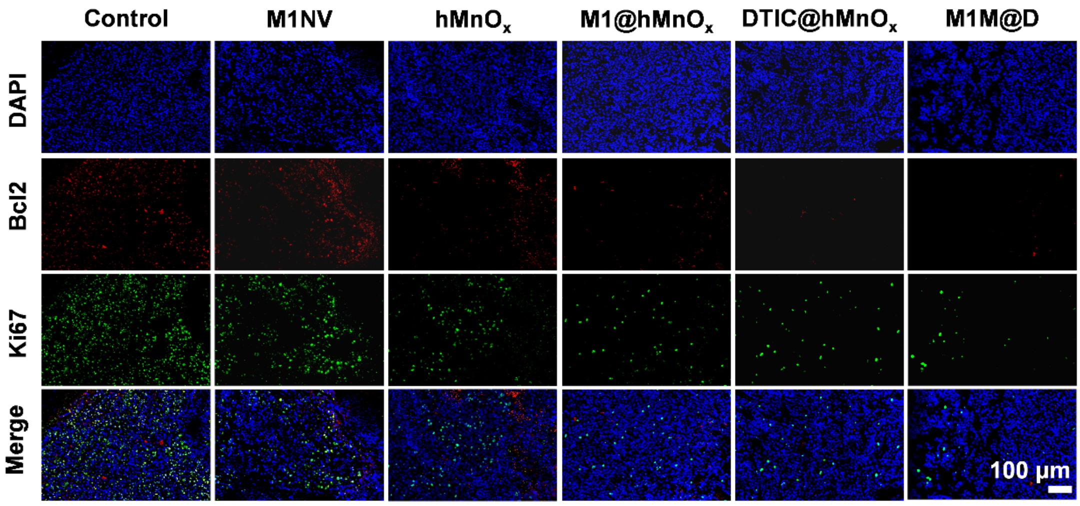

Figure S20. Immunofluorescence results of Ki67 and Bcl2 in tumor tissues.

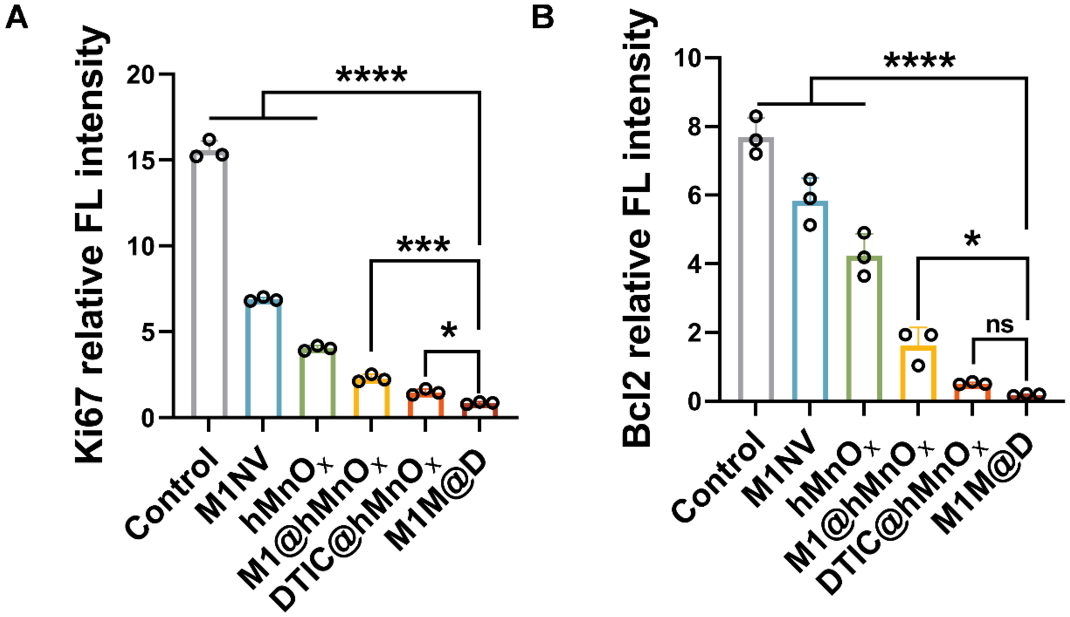

Figure S21. Relative FL intensity results of Ki67 and Bcl2 in tumor tissues. Data are presented as mean ± SD, $n = 3$. ns, non-significant ($p > 0.05$), * $p < 0.05$, ** $p < 0.01$, *** $p < 0.001$, **** $p < 0.0001$.

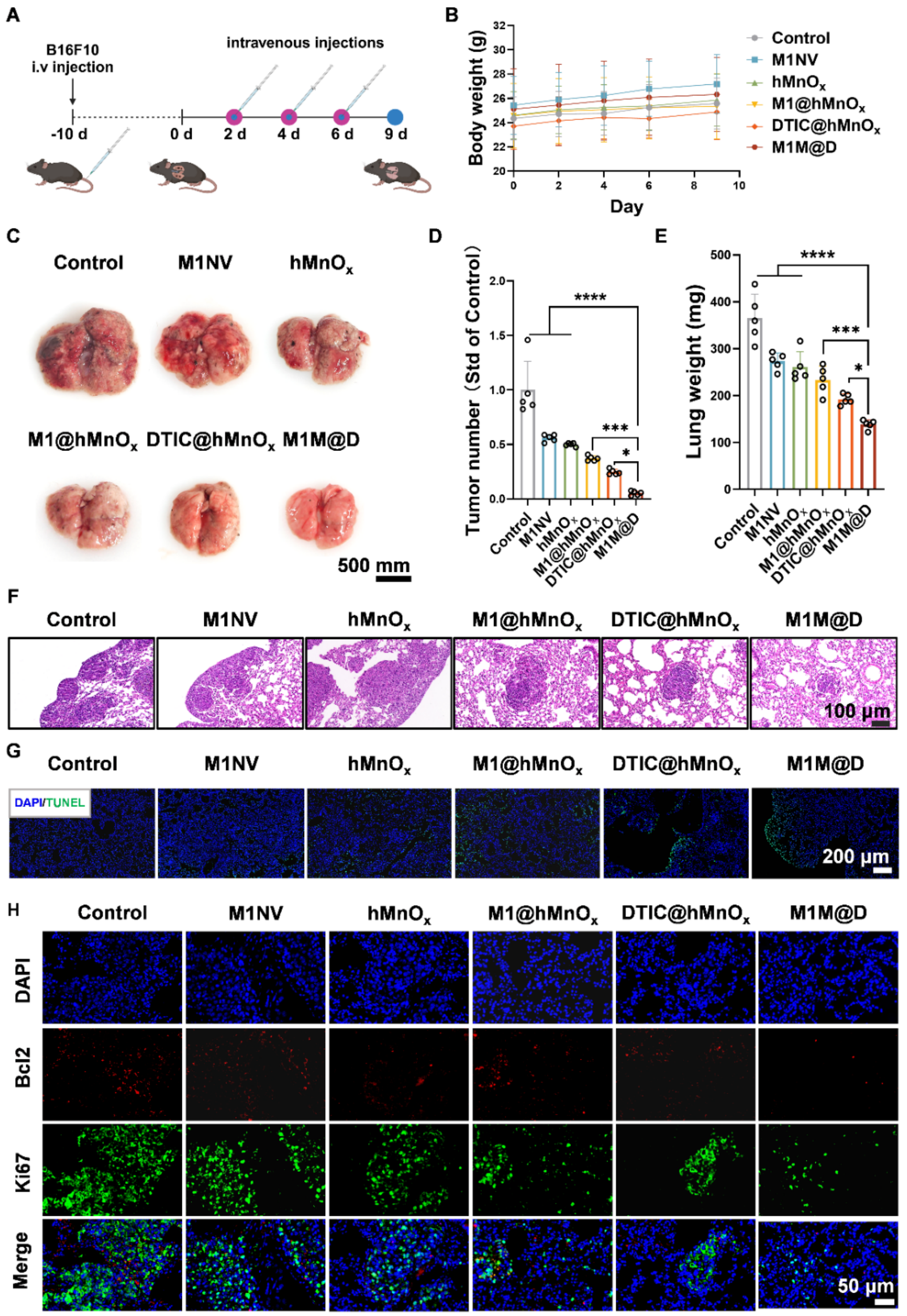

Figure S22. **Anti-tumor efficiency of M1M@Ds in lung metastatic tumor mode.** (A) Schematic diagram of the treatment protocol. (B) Body weight changes of mice in each treatment group. (C) Anatomy of lung tumor. (D) Tumor number for the tumors in each treatment group. (E) Lung weight for the tumors in each treatment group. (F) H&E and (G) TUNEL staining analyses of the tumors of mice in each treatment group. (H) Immunofluorescence results of Ki67 and Bcl2 in lung tumor tissues. Cell nuclei were stained with DAPI (blue). Data are presented as mean ± SD, n = 8. * $p < 0.05$, ** $p < 0.01$, *** $p < 0.001$, **** $p < 0.0001$.

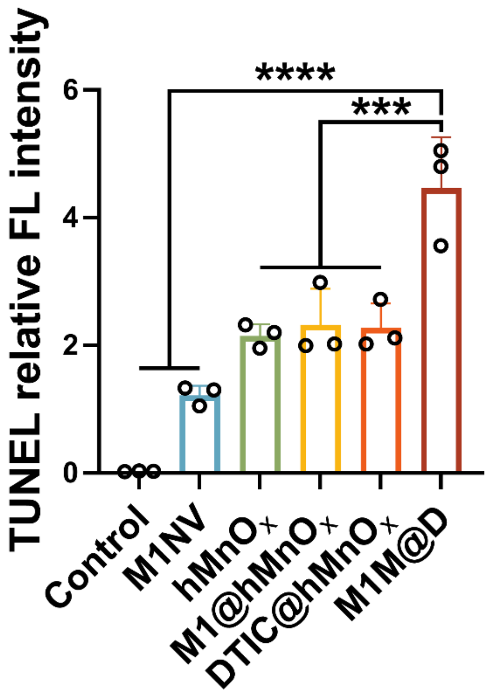

Figure S23. Relative FL intensity results of TUNEL in lung tumor tissues. Data are presented as mean ± SD, n = 3, *** p < 0.001, **** p < 0.0001.

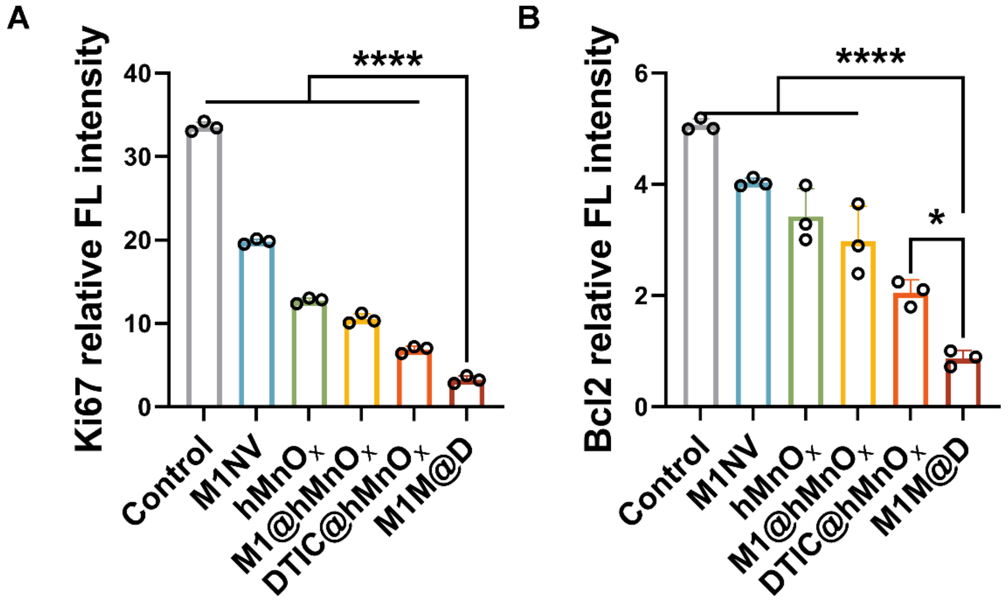

Figure S24. Relative FL intensity results of Ki67 and Bcl2 in lung tumor tissues. Data are presented as mean ± SD, n = 3, **** $p < 0.0001$.

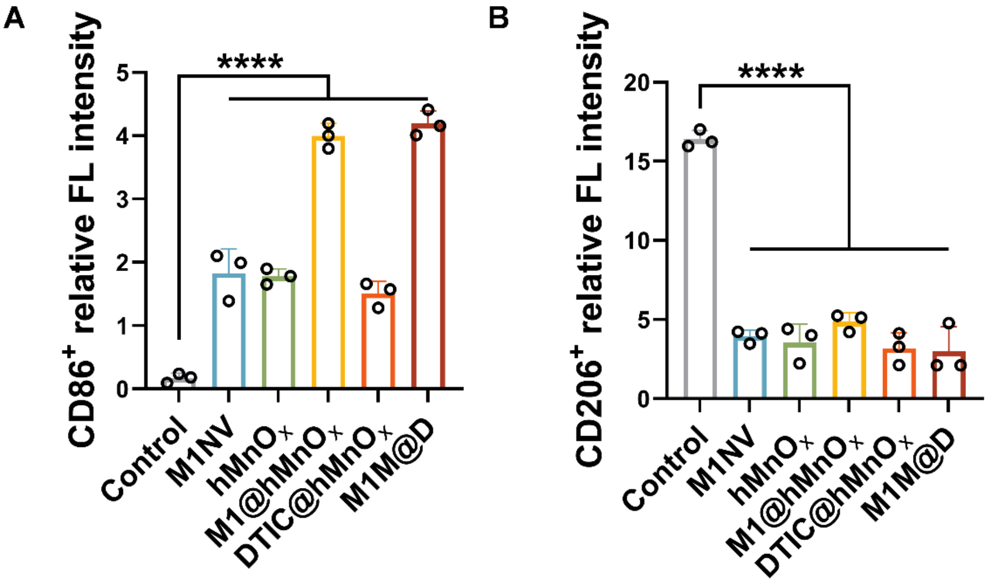

Figure S25. Relative FL intensity results of CD86$^+$ and CD206$^+$ cells in tumor tissues. Data are presented as mean ± SD, $n = 3$, **** $p < 0.0001$.

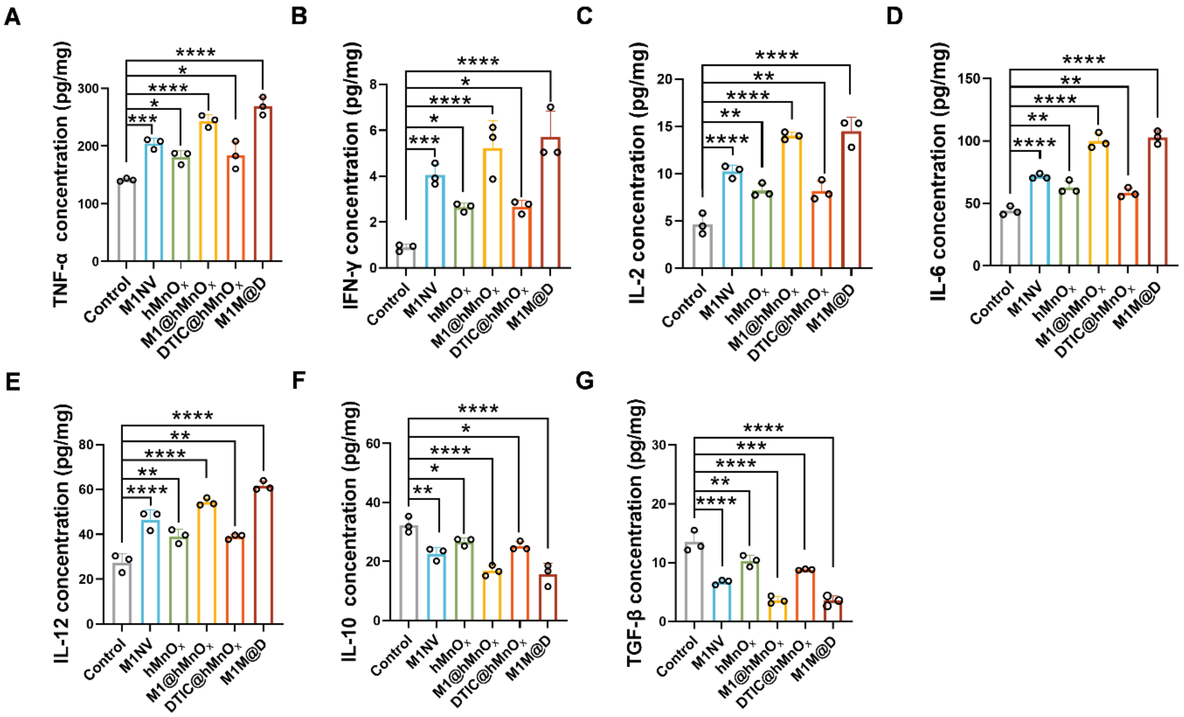

Figure S26. Secretion levels of (A) TNF-α, (B) IFN-γ, (C) IL-2, (D) IL-6, (E) IL-12, (F) IL-10 and (G) TGF-β in tumors after different treatments.

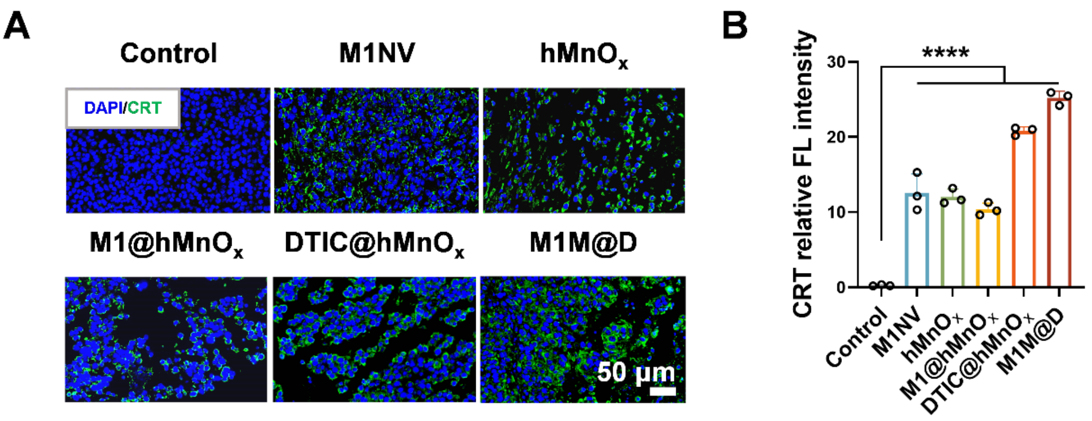

Figure S27. (A) Immunofluorescence staining images and (B) Relative FL intensity results of CRT in tumor tissues. Data are presented as mean ± SD, $n = 3$， **** $p < 0.0001$.

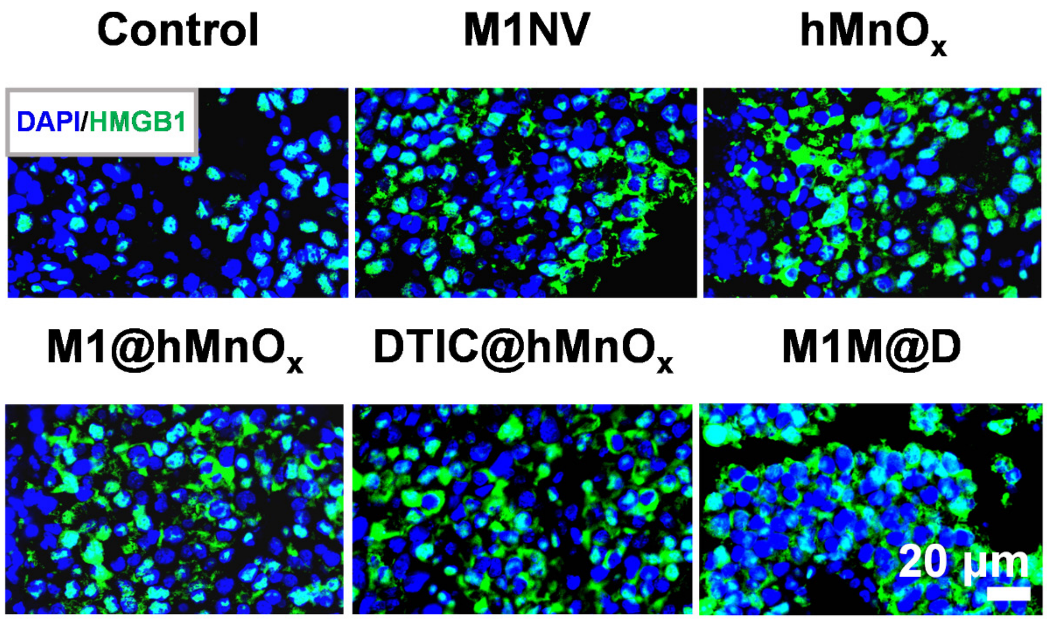

Figure S28. Immunofluorescence staining images of HMGB1 in tumor tissues.

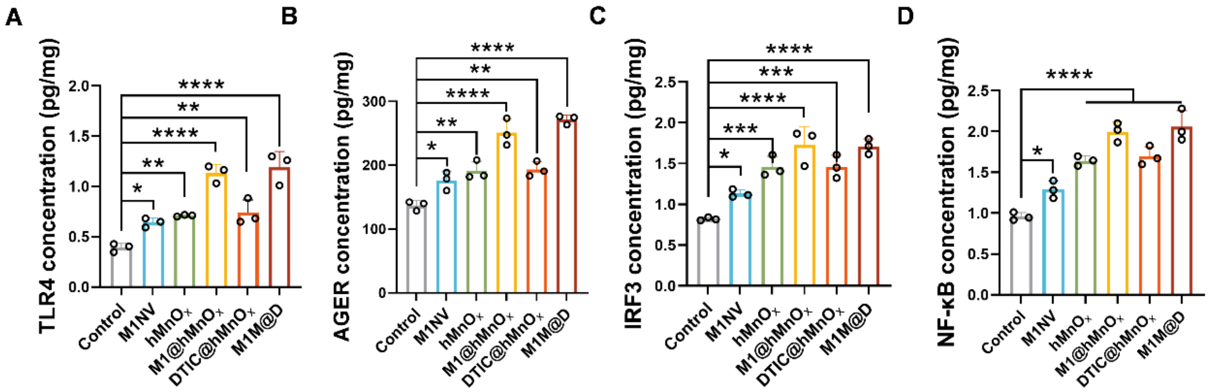

Figure S29. Secretion levels of (A) TLR4, (B) AGER, (C) IRF3, and (D) NF-κB in tumors after different treatments.

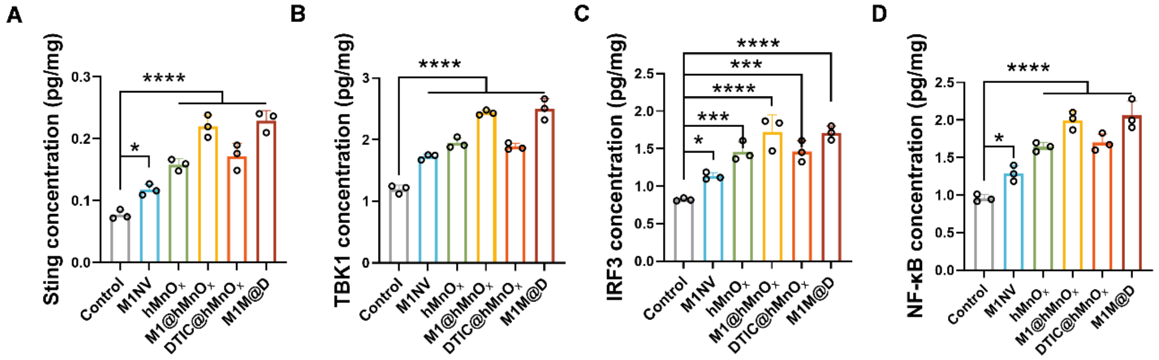

Figure S30. Secretion levels of (A) Sting, (B)TBK1, (C) IRF3, and (D) NF-κB in tumors after different treatments.

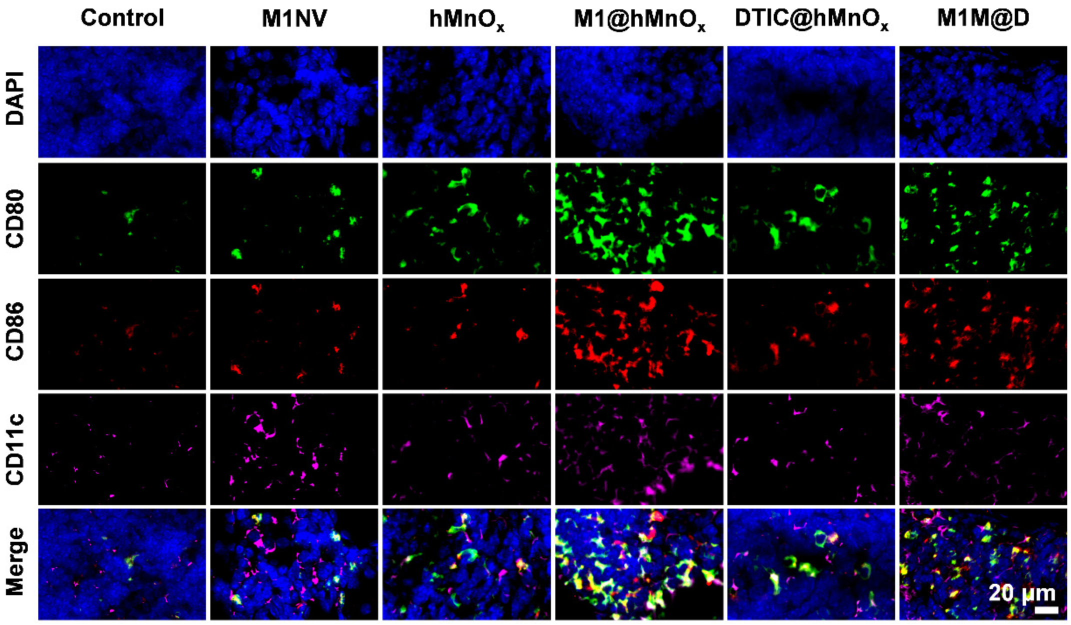

Figure S31. Representative immunofluorescence staining images of CD80$^+$, CD86$^+$, and CD11c$^+$ DCs in tumor draining lymph nodes.

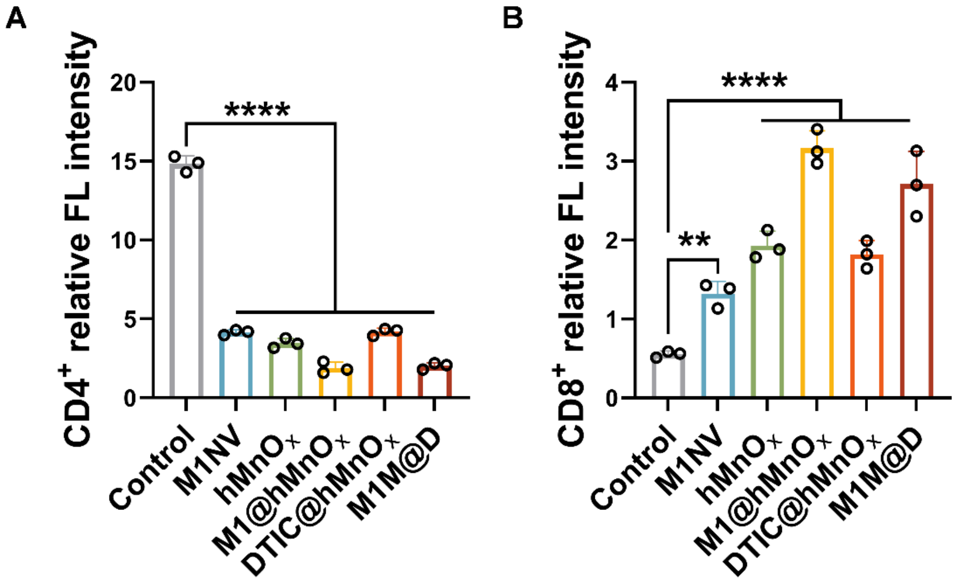

Figure S32. Relative FL intensity results of CD4$^+$ and CD8$^+$ T cells in tumor tissues. Data are presented as mean ± SD, $n = 3$, *** $p < 0.001$, **** $p < 0.0001$.

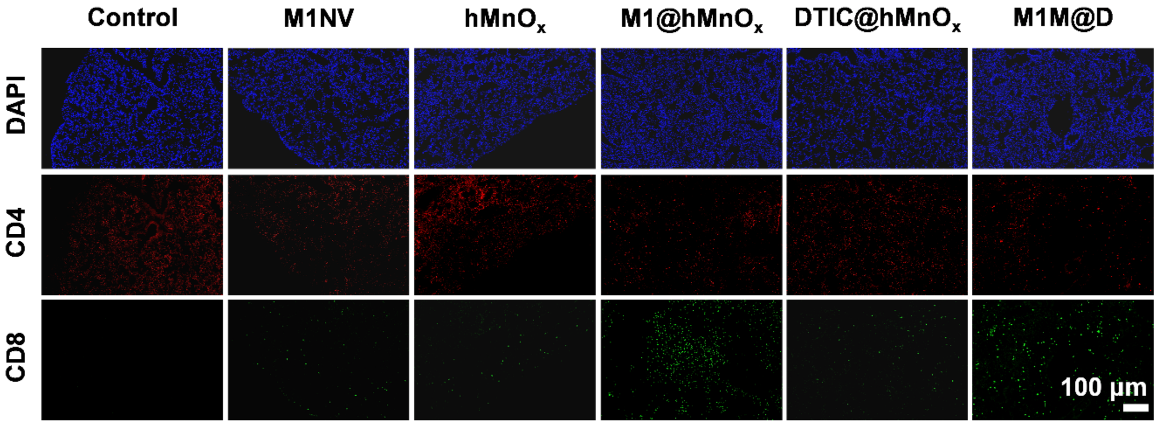

Figure S33. Immunofluorescence results of $CD4^+$ and $CD8^+$ in lung tumor tissues.

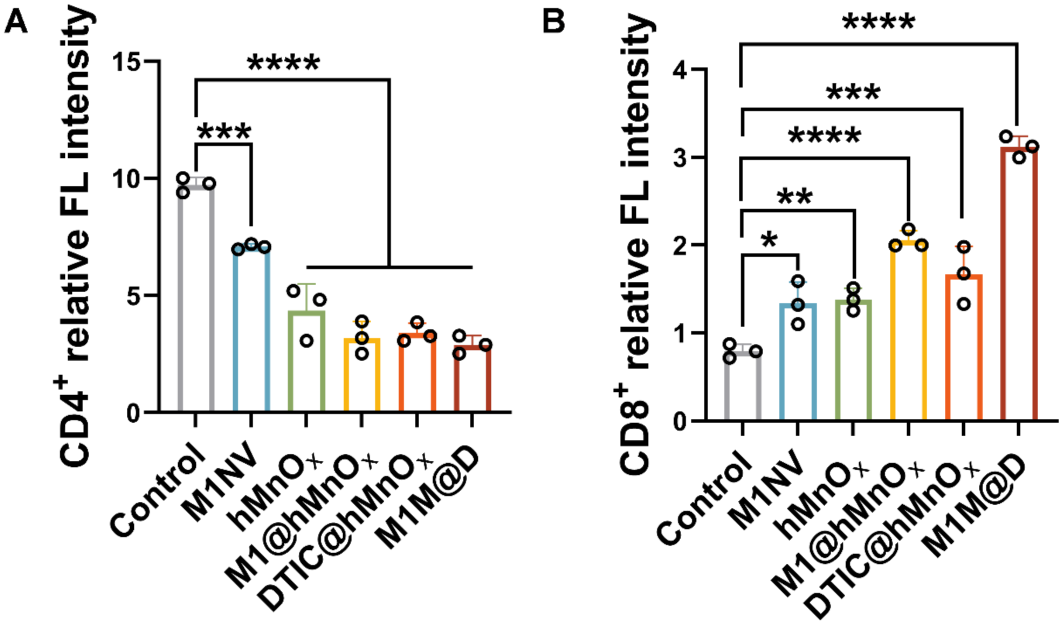

Figure S34. Relative FL intensity results of $CD4^+$ and $CD8^+$ in lung tumor tissues. Data are presented as mean ± SD, $n = 3$， * $p < 0.05$, ** $p < 0.01$, *** $p < 0.001$, **** $p < 0.0001$.

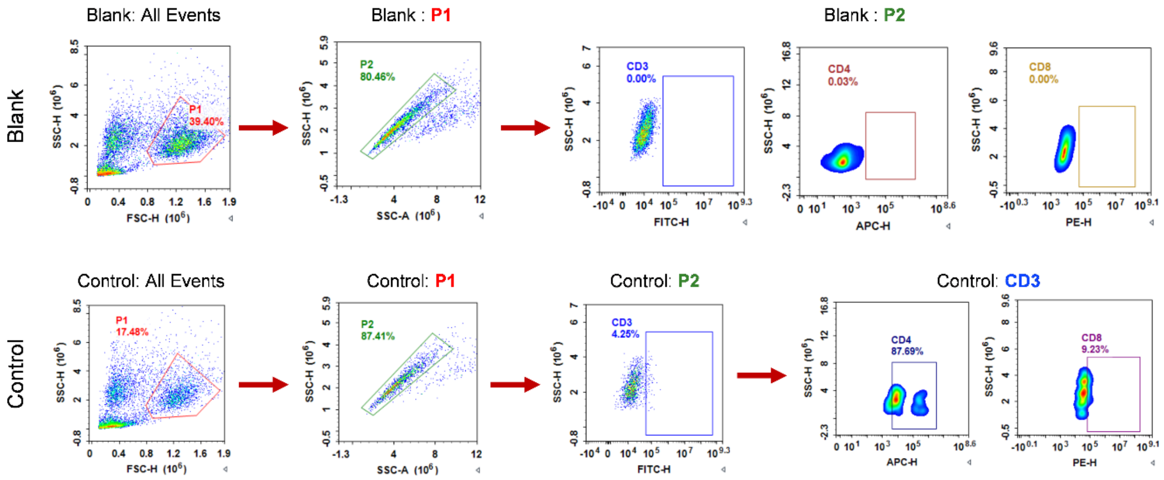

Figure S35. The gating strategy for $CD4^+$ and $CD8^+$ flow cytometry.

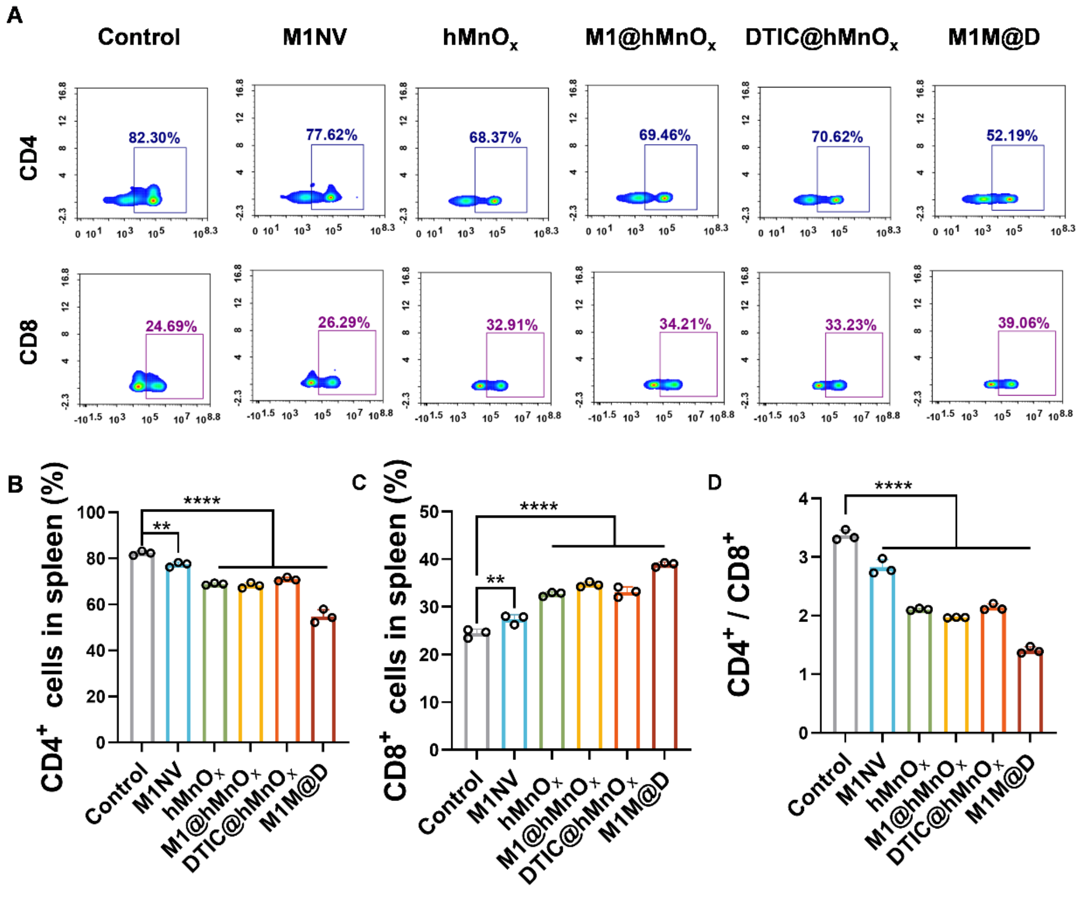

Figure S36. Flow cytometry results of $CD4^+$ and $CD8^+$content in spleen tissue. **(A)** The results of FCM of $CD4^+$ and $CD8^+$ in the spleen tissue. The quantifcation of the portion of **(B)** $CD4^+$, **(C)** $CD8^+$, and **(D)** the ratio of $CD4^+$ and $CD8^+$ subsets respectively. All data are expressed as mean ± SD, *n* = 3. On day 9, the tumor tissues were collected for analysis. * $p < 0.05$, ** $p < 0.01$, *** $p < 0.001$, **** $p < 0.0001$.

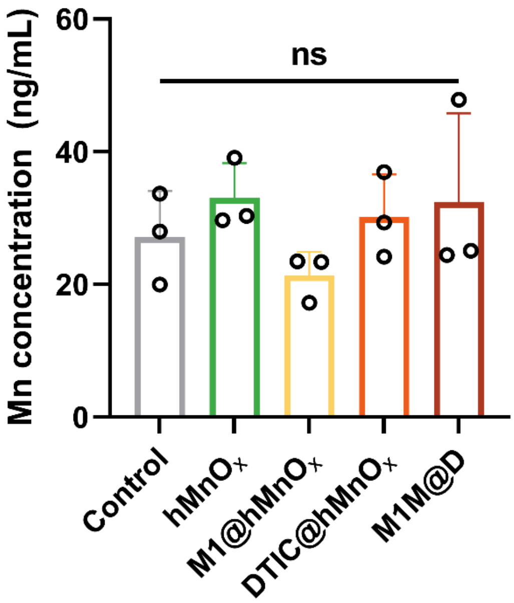

Figure S37. ICP-MS results of manganese ions concentration in brain tissues. Data are presented as mean ± SD, n = 3， ns (not significant, $p > 0.05$).

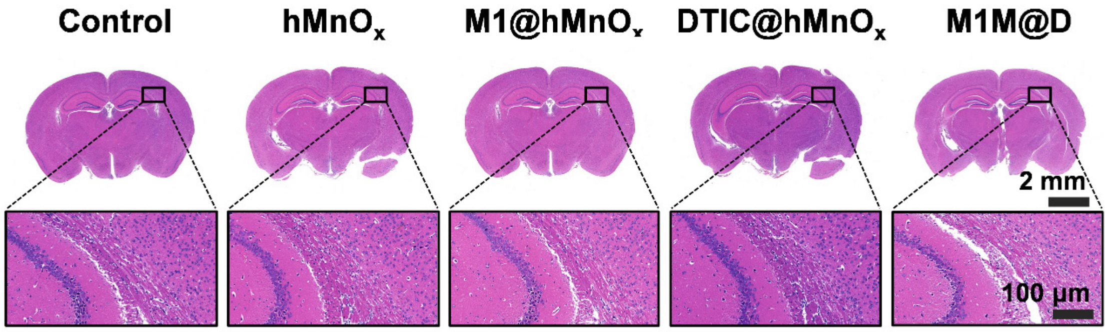

Figure S38. H&E of the brain tissues of healthy mice in each treatment group.

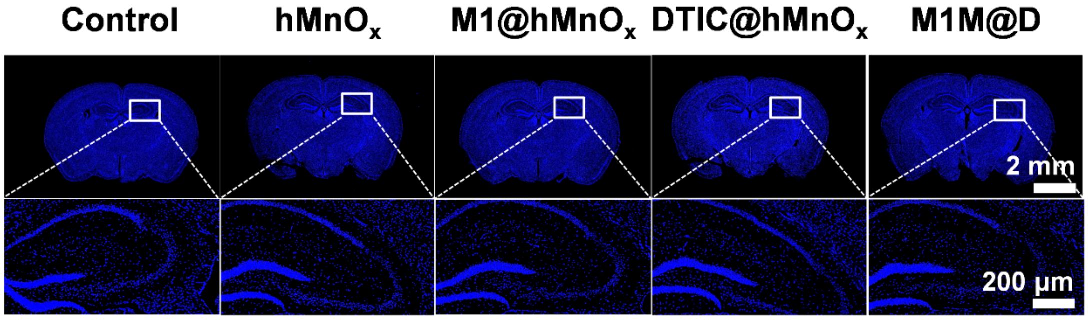

Figure S39. TUNEL staining analyses of the brain tissues of healthy mice in each treatment group.
